# Co-feeding of vacuum gas oil and pinewood-derived hydrogenated pyrolysis oils in a fluid catalytic cracking pilot plant to generate olefins and gasoline

**DOI:** 10.12688/openreseurope.14198.1

**Published:** 2021-11-30

**Authors:** Marco Buechele, Helene Lutz, Florian Knaus, Alexander Reichhold, Robbie Venderbosch, Wolfgang Vollnhofer

**Affiliations:** 1 Institute of Chemical, Environmental and Bioscience Engineering, Vienna University of Technology, Vienna, 1060, Austria; 2 BTG Biomass technology Group B.V., Enschede, 7521, The Netherlands; 3 R-T Business Transformation, OMV Downstream GmbH, Vienna, 1020, Austria

**Keywords:** Fluid Catalytic Cracking, vacuum gas oil, pyrolysis, co-feeding, hydrogenated pyrolysis oil, pinewood, olefins, high-octane gasoline

## Abstract

**Background:** The Waste2Road project exploits new sustainable pathways to generate biogenic fuels from waste materials, deploying existing industrial scale processes. One such pathway is through pyrolysis of wood wastes.

**Methods:** The hereby generated pyrolysis liquids were hydrogenated prior to co-feeding in a fluid catalytic cracking (FCC) pilot plant. So-called stabilized pyrolysis oil (SPO) underwent one mild hydrogenation step (max. 200 °C) whereas the stabilized and deoxygenated pyrolysis oil (SDPO) was produced in two steps, a mild one (maximum 250 °C) prior to a more severe process step (350 °C).

These liquids were co-fed with vacuum gas oil (VGO) in an FCC pilot plant under varying riser temperatures (530 and 550 °C). The results of the produced hydrocarbon gases and gasoline were benchmarked to feeding pure VGO.

**Results: **It was proven that co-feeding up to 10 wt% SPO and SDPO is feasible. However, further experiments are recommended for SPO due to operational instabilities originating from pipe clogging. SPO led to an increase in the hydrocarbon gas production from 45.0 to 46.3 wt% at 550 °C and no significant changes at 530 °C. SDPO led to a rise in gasoline yield at both riser temperatures. The highest amount of gasoline was produced when SDPO was co-fed at a 530 °C riser temperature, with values around 44.8 wt%. Co-feeding hydrogenated pyrolysis oils did not lead to a rise in sulfur content in the gasoline fractions. The highest values were around 18 ppm sulfur content. Instead, higher amounts of nitrogen were observed in the gasoline.

**Conclusions: **SPO and SDPO proved to be valuable co-refining options which led to no significant decreases in product quality. Further experiments are encouraged to determine the maximum possible co-feeding rates. As a first step, 20-30 wt% for SPO are recommended, whereas for SDPO  100 wt% could be achievable.

## Plain language summary

To reduce the effect of climate change, alternatives to fossil fuels are investigated. One close-to-market option is to use liquids derived from biogenic materials. These biogenic wastes are a great resource since they are comparably cheap and do not compete with food/feed production.

The liquid products used here were produced through fast pyrolysis of woody biomass. They were then further improved using hydrogenation to make them easier to handle for fuel production. The improved liquids were fed together with standard fossil oils in a process called fluid catalytic cracking (FCC). This FCC process is a standard process in many refineries. The main products of the FCC process are gasoline and olefins, which are gases that can be used to create plastics.

In this paper we screened the quality of waste-derived products while co-feeding them into such an FCC unit. Product yields and compositions were compared to those obtained when only fossil oils are used as feedstock.

## Introduction

In the 2015 Paris climate agreement, the international community declared that net CO2 emissions need to be reduced, and eventually eliminated to keep global warming below 1.5 °C ^1^. Based on that, the European Union has communicated their long-term plan of fulfilling this goal by creating a path to reach net zero emissions by 2050 ^2^. “WASTE2ROAD – Biofuels from Waste to Road transport” is funded through the European Framework Programmme for Research and Innovation Horizon 2020 and aims to develop new cost-effective biofuels out of residues and waste streams. Value chains, from waste management to vehicle tests of the created biofuels, shall be established partly using pre-existing refinery technology ^3^. One of the key refinery processes is fluid catalytic cracking (FCC), which is one of the main conversion processes in a refinery. It is a highly versatile process, enabling it to convert low-value feedstocks and heavy residues to produce high-octane gasoline, making it an ideal candidate for processing new alternative feedstocks ^4^. This versatility has been researched in the past with different alternative feedstocks like Fischer-Tropsch-waxes and vegetable oils ^5– 7^.

The use of biogenic material-derived liquids in FCC units with vacuum gasoil (VGO) is technically and economically feasible. Fast pyrolysis is a cost-effective and attractive depolymerization process applied to convert such biogenic materials into so-called pyrolysis liquids (PL). It operates under reasonable conditions (atmospheric pressure, maximum temperatures of 550 - 600 ^o^C) and high yields of PL (up to 70 wt%). Tests verified that co-processing of crude PLs is technically possible. However, a different behavior of the liquids is noted, especially when comparing results from small batch systems and bigger continuously operated units ^8, 9^.

Large amounts of oxygenated compounds make up the PL which originate from the utilized feedstock and, as a consequence, restrict the feeding of such pure liquids in FCC units. In most studies, low overall (renewable) carbon yields have been noted, along with, or perhaps because of, severe operational issues. At the expense of the gasoline fraction, high coke and dry gas yields have been reported. This is due to the reactivity of the carbohydrate fraction in these liquids. Consequently, a pre-processing step, such as hydrogenation, benefits co-processing of such low-quality liquids.

A first approach was proposed at the end of the 1990’s: upgraded liquids were successfully co-processed with crude oil derivatives with similar yields of the FCC products as with pure VGO. ^10^. The hydrogen consumption is further explored to improve the PLs quality, aiming at more optimal economic conditions for co-processing, while simultaneously addressing the quality of the products ^11^. Overall, only minor pre-processing of the liquids is required to avoid significant operational problems and reduce the coke formation, resulting in higher gasoline yields.

The present work proposes to co-process such pretreated liquids from biogenic materials, a mildly and a more severely hydrotreated liquid, here respectively referred to as SPO and SDPO. Both liquids were fed at a constant substitution level of up to 10 wt% bio-liquids. Data were produced on a pilot-scale continuously operated unit, closely resembling data derived in commercial units.

## Methods

### Pilot plant

**Figure 1.**
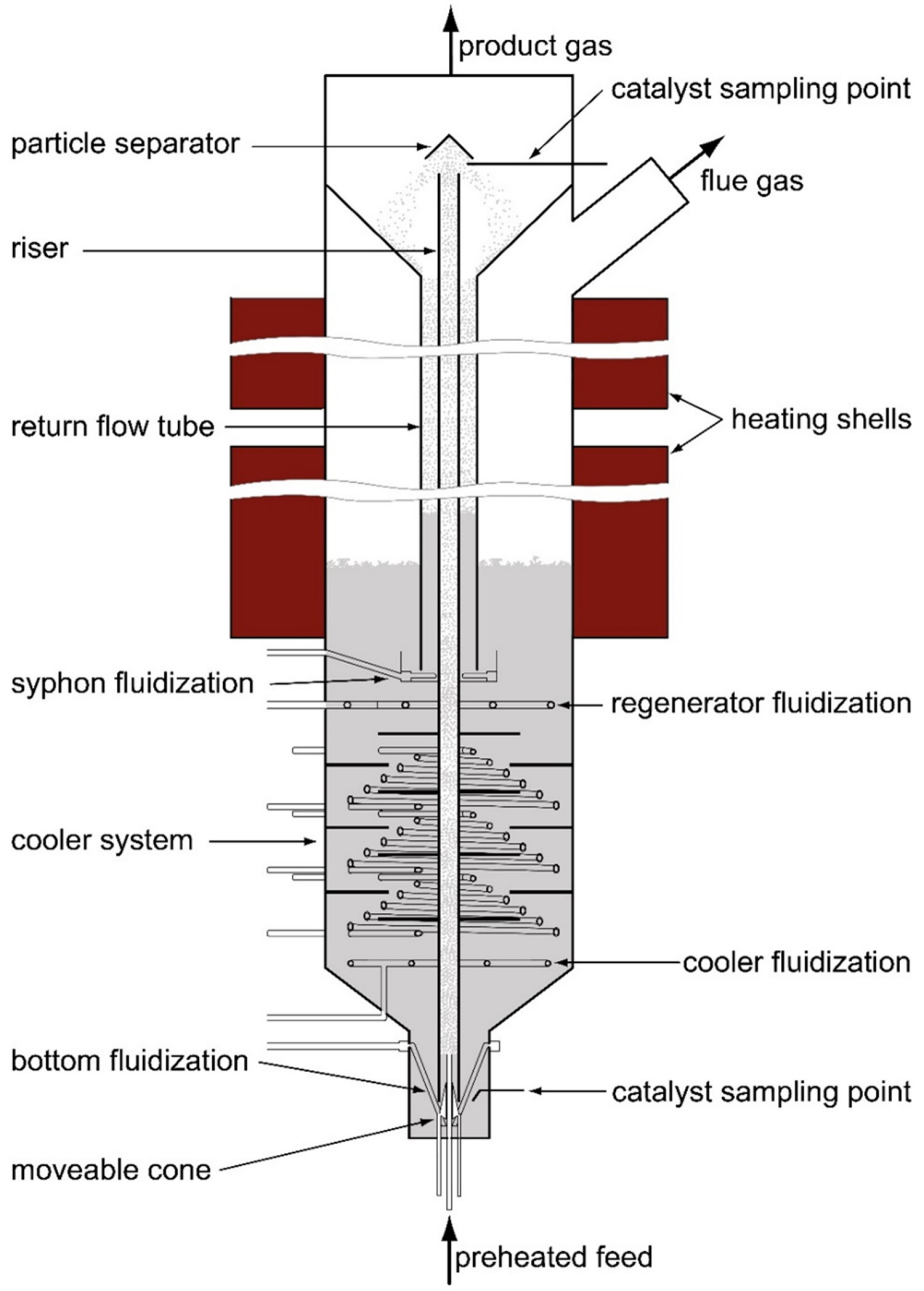
Pilot plant.

The continuously operated FCC pilot plant was an internally circulating fluidized bed system located at the Institute of Chemical, Environmental and Bioscience Engineering (ICEBE) at Technical University Wien (TUW). This means that the regenerator and riser section were located in the same column, resulting in a stronger thermal coupling of the two sections compared to industrial FCC plants with external circulation. A positive side effect of this is the plant’s compact design. A schematic of the plant is depicted in Figure 1. The key pilot plant data is summarized in Table 1.

**Table 1.** Key data pilot plant.

Total height	3.2 m
Riser length	2.5 m
Riser diameter	0.0215 m
Regenerator diameter	0.33 m
Regenerator temperature	500 - 800 °C
Riser temperature	400 - 700 °C
pressure	atmospheric
Catalyst mass	45 - 75 kg
feed rate	1,5 - 8 kg/h
Riser residence time	~̴1 s
catalyst circulation rate	0.5 - 5 kg/min
C/O-ratio	10 - 50

The feed was pumped via a gear pump into the riser passing through a tubular oven, where it was heated close to boiling temperature. As soon as the feed entered the reactor section (riser) it got in contact with the hot catalyst. Thus, the feedstock immediately evaporated, leading to a greater volume flow by several orders of magnitude. This increase in volume accelerated the upwards rising motion of the feed and catalyst particles, hence the reactor section being called a “riser”. During this upward motion cracking reactions occurred, and after a residence time of around one second the mixture reached the end of the riser. There, the catalyst was separated from the cracking gases using a particle separator. Additionally, an increase in diameter at the plant top, which resulted in a reduction of flow velocity, supported gas and particle separation. Thanks to that, over-cracking of the feed was prevented. The cracking product left the plant through the top while the catalyst particles fell down the return flow tube. They entered the regenerator through a syphon. There, deposited coke, which is generated as a byproduct, was burned off the catalyst, thereby reactivating the catalyst. The regenerated catalyst then flowed through the cooling section to the bottom. There, it was sucked again into the riser. This closed cycle enabled a continuous plant operation.

Nitrogen was used as the fluidization gas for plant operation, except for the regenerator and cooler section, which were fluidized with air. This was especially important for the bottom section and the syphon, since they both act as a gas barrier preventing cracking gas from entering aerobe plant areas. The syphon also acted as a stripper, removing remains of cracking gases from the catalyst pores. The cooler section made it possible to operate the plant in a wide temperature range (see Table 1).

For further information about the pilot plant, complementary literature is recommended: The basic design of the first version of the pilot plant was done by Reichhold ^12^, while Bielansky ^13^ constructed a second pilot plant and further developed it into its current state. For this work, the latter was used.

Realizing that SPO was not miscible with VGO, two separate pumps were used for feeding SPO and VGO respectively. The SPO and VGO were mixed just before the tubular oven. For the SDPO/VGO mixtures, the co-feeding experiments could be conducted through one pump. For the pilot plant experiments, SDPO and VGO mixtures were prepared beforehand with the desired amount of SDPO. The desired admixture of SPO, on the other hand, was ensured by regulating the power outputs of the two utilized pumps.

### Feedstock and catalyst

***Hydrotreated bio-liquids.*** The specific nature and chemical structure of pyrolysis liquids result in their thermal instability and may cause undesired re-polymerization of organic matter. Their reactivity is not due to the relative amount of oxygen, but to the existence of highly reactive oxygen containing carbonyl groups, mainly in the form of aldehydes or ketones. Direct hydrogenation of these compounds and transformation into less reactive organic groups (i.e., into the corresponding alcohols) reduces this reactivity problem. General properties of different products and the associated processes applied in this work are summarized in Table 2.

**Table 2.** Main properties of hydrotreated pyrolysis liquids and associated processes. Products and processes relevant for this work have been highlighted. SPO: stabilized pyrolysis oil. SDPO: stabilized and deoxygenated pyrolysis oils

Product	Properties			Process		
	Water content	Oxygen	MicoCarbon residue testing result	Catalyst	Temperature	Pressure
	[wt%]	[wt%]	[wt%]	-	[°C]	[Bar]
Pyrolysis oil	> 18	> 35	> 15	-	-	-
SPO	< 5	15-35	5-20	Picula ^TM^	80-250	200
SDPO	< 1	< 15	< 5	CoMo/NiMo	> 300	200

For the present purpose, the stabilization of the pyrolysis liquids was carried out in a single-step process, in which around 10 kg of liquid were processed. A packed bed reactor system was applied, with four separate reactors each containing around 30-35 g catalysts. Prior to the experiment, the catalyst was activated in the presence of hydrogen, at elevated temperature (600 ^o^C) and atmospheric pressure. The conditions applied and resulting flow rates are shown in Table 3.

**Table 3.** Process conditions in the SPO benchmarking experiment.

Temperature [°C]				Pressure [bar]	Average liquid flow [g/h]	Average hydrogen flow [Nl/h]
Reactor 1	Reactor 2	Reactor 3	Reactor 4			
75	100	140	200	200	80	30

At these conditions, the temperatures in the first three reactors were stable, indicating that the heat of hydrogenation reactions was low, but a notably mild temperature excursion from 200 – 250 ^o^C was observed in the fourth reactor as a result of the hydrogenation reactions. Part of the SPO was further deoxygenated. 

The deoxygenation of these SPO liquids was also carried out as a single-step process, in a similar set-up as for the SPO but at elevated temperatures, in which over 5 kg of SDPO were produced.

Each of the reactors was filled with around 25 g of commercially applied NiMo catalyst. Prior to the experiment, the catalyst was activated in the presence of hydrogen and diesel with a minor amount of dimethyl disulfide (DMDS) from Sigma-Aldrich as a sulfur precursor, at temperatures up to 350 ^o^C and a processing pressure of 200 bar. The operating conditions and resulting flow rates for the SDPO production process are shown in Table 4.

**Table 4.** Process conditions in the SDPO benchmarking experiment.

Temperature [°C]				Pressure [bar]	Average liquid flow [g/h]	Average hydrogen flow [Nl/h]
Reactor 1	Reactor 2	Reactor 3	Reactor 4			
200	250	300	350	200	100	60

By hydrotreating, it is thus possible to obtain a high quality, oxygen-free product. However, this requires a long processing time, high energy and hydrogen consumption, and leads to a lower overall yield of the whole value chain from biomass to refined bio-oil.

**Figure 2.**
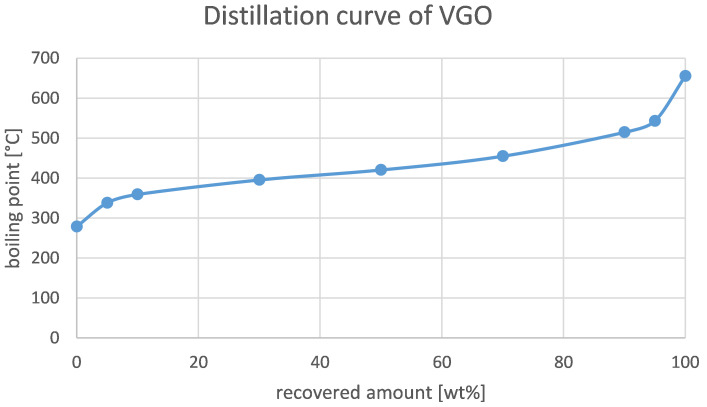
Distillation curve of VGO.

***Vacuum gas oil.*** VGO is a standard feedstock for industrial FCC plants and is the top product of the vacuum distillation ^14^. It was provided by Austrian oil and gas company OMV and originates from the OMV refinery in Schwechat, Austria. Some key parameters of the utilized VGO batch are listed in Table 5. The distillation curve is depicted in Figure 2. There, it is observed that evaporation started around 280–290°C and that the final boiling point was around 650°C.

**Table 5.** Parameters of VGO.

parameter	value	unit
density at 15 °C	885	kg/m³
viscosity at 100 °C	5.584	mm²/s
total sulfur	291	mg/kg
total nitrogen	275	mg/kg
nickel	2	mg/kg
vanadium	2	mg/kg
Conradson carbon residue	0.294	wt%
total aromatics	23.8	wt%

The catalyst was obtained from the OMV Schwechat refinery and was therefore an equilibrium catalyst (E-cat). This was done to provide maximum comparability between the industrial-scale plant and the pilot plant. The catalyst was a mixture of two commercially available catalysts that were designed for enhanced production of propylene and butanes/butenes. They were both based on zeolites and enhanced with rare earth oxides. Additional information regarding the catalyst cannot be disclosed since there is no permission of the catalyst vendor.

### Analysis

In cracking processes, wide varieties of hydrocarbons are generated, leading to the necessity of developing an analysis model that provides enough detail with reasonable effort. In FCC processes, one often used model is the so-called lump model, in which the products are categorized in different groups or lumps. Each lump is defined by its composition or its boiling range and gets measured by defined analysis methods. The lump model employed is described in Table 6.

**Table 6.** Lump model and corresponding analysis method.

Fraction	Product group	Boiling range, composition	Analysis method
**Gaseous**	Carbon oxides	CO, CO _2_	Nondispersive infrared spectroscopy
	Hydrocarbon gas	C1 – C4	Gas chromatography
**Liquid**	Gasoline	< 210 °C	Simulated distillation
	Light cycle oil	210 – 320 °C	Simulated distillation
	Residue	>320 °C	Simulated distillation
	Water	H _2_0 + dissolved substances	Gravimetric
**Solid**	Coke	Burned-off substances	Flue gas composition

In each experiment, at least two, but in most cases three different samples of the gas product were taken for analysis. Each sample was collected over a period of 15 minutes and then analyzed at least twice.

The product consisted of three different phases (gas, liquid and solid). The gas phase was extracted in a gas collection tube and analyzed in a gas chromatograph from Shimadzu (model GC-17A). The configuration of the gas chromatograph is described in detail in Table 7. The gaseous hydrocarbons were separated in a VARIAN CP-Al _2_O _3_/Na _2_SO _4_-column, and determined with a flame ionization detector that measures all C _1_-C _4_ hydrocarbons. In a second measuring section, the amount of fluidization gas (nitrogen) in the gas product was determined using a CP carboPLOT P7 column and a thermal conductivity detector. The carbon oxides (CO _x_) were measured online using an infrared gas analyzer (NGA 2000 MLT 3 from Emerson).

**Table 7.** Gas-GC configuration.

Gas-GC Shimadzu GC-17 A	
**Injector**	Splitless 50 µl at 200 °C
**Carrier gas**	Helium 1.46 ml/min constant flow
**Temperature program**	50 °C to 200 °C; dwell-time 30 min
**Phase**	I: 100% Polydimethylsiloxan II: Carbon Porous Layer
**Columns**	I: Varian CP-Al _2_O _3_/Na _2_SO _4_ II: CP CarboPLOT P7
**Dimensions**	I: 50 m x 0.25 mm ID x 4 µm II: 27.5 m x 0.53 mm ID x 25 µm
**Detectors**	I: Flame Ionization Detector (FID) at 200 °C II: Thermal Conductivity Detector (TCD) at 105 °C

The liquid fraction consisted of an organic and an aqueous phase that could easily be separated in a separation funnel. The water was measured gravimetrically. The organic phase was measured with a second gas chromatograph, using a simulated distillation (SimDist). The SimDist’s configuration is described in Table 8 and determined the probes’ composition regarding its boiling range. The organics were hereby separated in gasoline, light cycle oil (LCO) and residues.

**Table 8.** SimDist-GC configuration.

SimDist-GC Shimadzu GC-17A	
**Injector**	Split 30:1 1.5 µl at 350 °C
**Carrier Gas**	Hydrogen 1.68 ml/min constant flow
**Temperature program**	35 °C to 350 °C; dwell-time 22 min
**Phase**	100 % Polydimethylsiloxane
**Column**	Zebron ZB-1
**Dimension**	30m x 0.32mm ID x 0.25 µm df
**Detector**	Flame Ionization Detector (FID) at 350 °C

The gasoline lump of the organics fraction was manually distilled in a laboratory distillation column. The cut-off temperature of 210 °C was chosen since it is the dictated final boiling point of gasoline according to norm DIN EN 228:2012 ^15^. This gasoline fraction was sent to the OMV laboratory for further analysis of basic properties such as density, dissolved water content, elemental analysis (CHNSO), boiling curve (SIMDIS), Reid vapor pressure (RVP), PIONA (paraffin, isomer, olefin, naphthene and aromatic content), inorganic contaminants (ICP-MS+Chlorides), acid value and oxidation stability. The OMV technical laboratory does not want to disclose the exact applied measurement procedures (equipment and norms) for confidentiality purposes.

The solid fraction, the coke, was determined indirectly. The burned off coke in the regenerator was converted into CO, CO _2_ and H _2_O that are found in the flue gas. Through measurements of CO, CO _2_ and O _2_, the coke amount could be computed using combustion calculation. Again, CO, CO _2_ and O _2_ were determined via online gas analyzers as described before.

Overall conversion is an indicator of the process’ economic viability. In this work, it is defined as the sum of gas and gasoline over the amount of feed. LCO is sometimes included in the conversion, but is often not considered a valuable product, even though it is in the distillation range of diesel and heating oil. This is due to its high aromatics content. Therefore, LCO is mostly fed back into the plant as a feedstock to be cracked again, hence its name, light cycle oil ^16^.


$$Conversion=\;\frac{m_{hydrocarbon\;gas}+m_{gasoline}}{m_{feed}}$$

## Results and discussion

### Gaseous fraction

In total, 10 experiments were carried out (see Table 9). Two benchmarking experiments were conducted with pure VGO for comparison with both SPO and SDPO admixtures. Additionally, four experimental runs were conducted for each type of pyrolysis oil.

**Table 9.** Configurations of FCC pilot plant experiments; VGO = Vacuum gas oil, SPO = Stabilized pyrolysis oil, SDPO = Stabilized and deoxygenated pyrolysis oil.

Feedstocks	Admixtures [wt%]	Riser temperature [°C]
VGO	0	550
VGO	0	530
VGO + SPO	5	550
VGO + SPO	5	530
VGO + SPO	10	550
VGO + SPO	10	530
VGO + SDPO	5	550
VGO + SDPO	5	530
VGO + SDPO	10	550
VGO + SDPO	10	530

### FCC results for co-feeding of SPO with VGO

**Figure 3.**
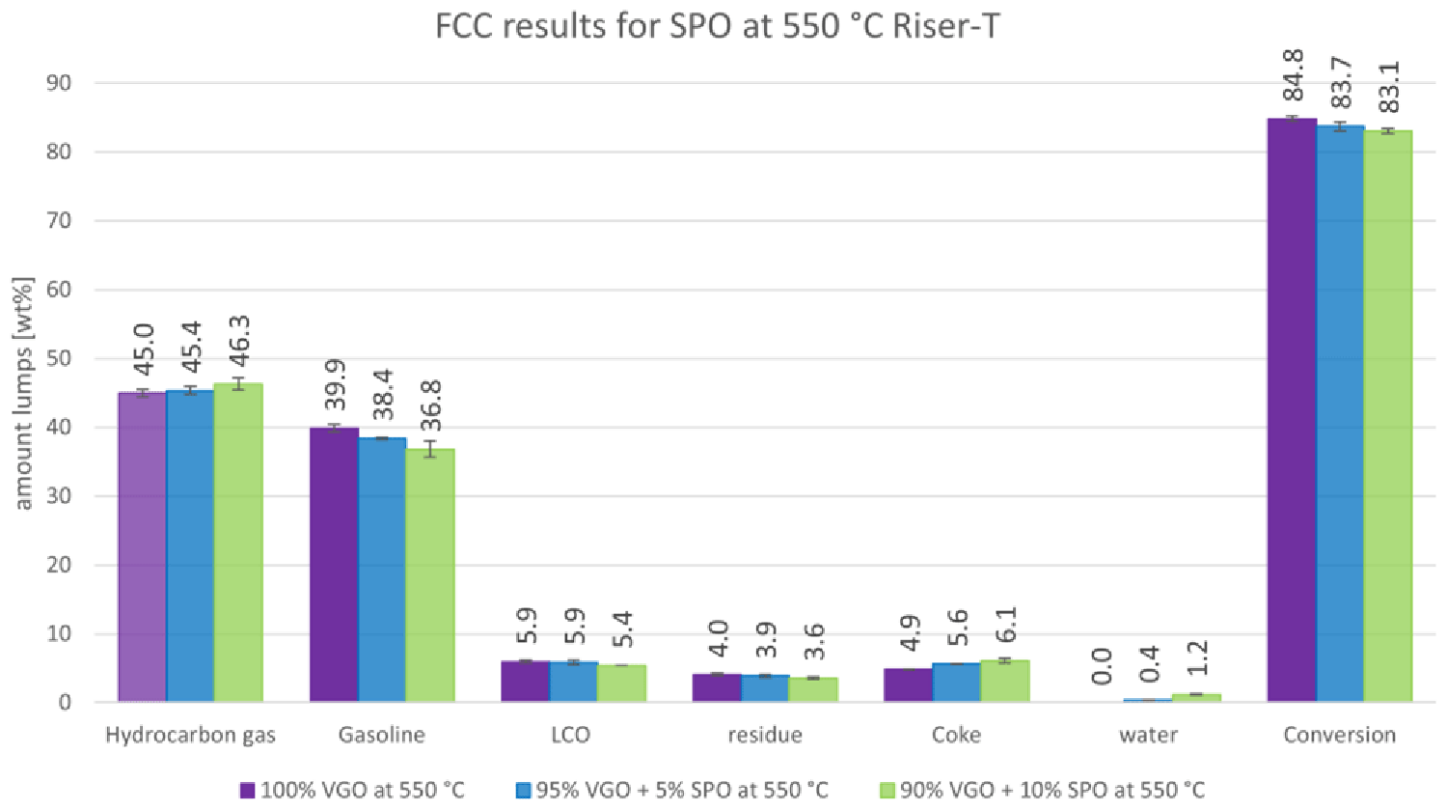
Product distribution depending on SPO admixture at 550 °C riser temperature.

**Figure 4.**
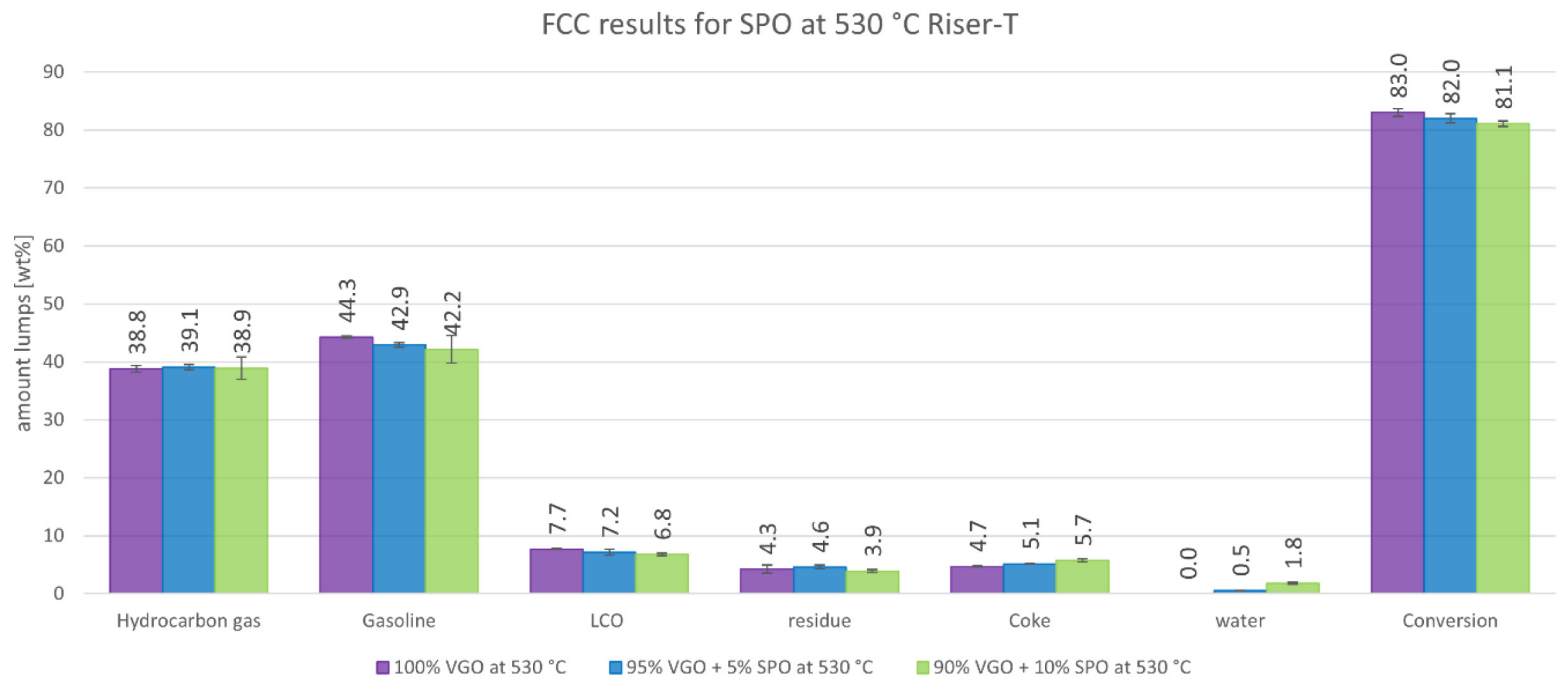
Product distribution depending on SPO admixture at 530°C riser temperature.

The product distributions according to the aforementioned lump model are depicted in Figure 3 and Figure 4. These figures show the change in product distribution depending on the amount of SPO that was co-fed into the plant. Figure 3 illustrates the change at a 550°C riser temperature whereas Figure 4 shows the changes at 530°C.

Figure 3 shows that a higher amount of SPO led to a higher gas formation and lower gasoline yields. The gas amount rose from 45.0 to 46.3 wt% while the gasoline lump decreased from 39.9 to 36.8 wt%. Additionally, the other liquid products, LCO and residue, declined from 5.9 to 5.4 and 4.0 to 3.9 wt%, respectively. The coke amount increased significantly from 4.9 to 6.1 wt%, due to the higher tendency of SPO to form coke. The water amount also increased from 0 to 1.2 wt%, since no water and oxygen are present in VGO, whereas SPO has a significant amount of both. The conversion slightly decreased from 84.8 to 83.1 wt%. All these changes were significant, according to the error margins. It must be mentioned, however, that higher amounts of SPO led to a wider variety of results due to an increasing operation instability of the pilot plant.

The results in Figure 4 for SPO at 530°C show a similar trend compared to 550°C. However, the liquid products (gasoline, LCO, residue and water) were significantly higher, which was expected since lower cracking temperatures lead to fewer cracking reactions, and therefore generate a higher yield of higher molecular weight molecules. The gas and coke fraction was also lower for the same reasons. The conversion was slightly lower ranging from 83.0 to 81.1 wt%.

Higher instability during operation when co-feeding 10 wt% SPO was observed, which was also indicated by slightly higher error margins in the measurements. Therefore, the influence of co-feeding SPO at a 530°C riser temperature is not clearly determined for all products. It is expected however that at 530°C, the amount of gas also increases at the expense of gasoline if SPO is co-fed (according to the results at 550°C). Further experiments must be carried out with SPO admixtures to confirm this hypothesis. However, a different feeding approach, in which SPO and VGO are fed separately in two independent lines should be considered. This approach would prevent pipe clogging caused by the coking of SPO and create a more stable plant operation.

**Figure 5.**
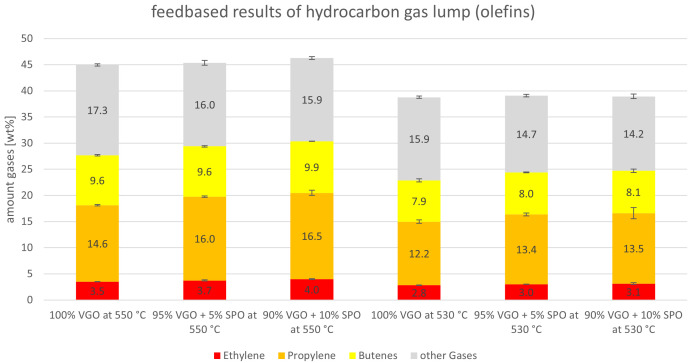
Olefins and other gases (alkanes) depending on riser temperature and SPO admixture.

The yields of hydrocarbon gases (olefins as ethylene, propylene and butenes) are depicted separately in Figure 5. Alkanes are grouped together here as “other gases”. Carbon oxides are not included in this graph. In Figure 5, the results for both riser temperatures are included. Here, the experiments at a 550°C riser temperature show generally higher values than the ones at 530°C, which was expected since higher cracking temperatures lead to higher gas yields. A positive finding is that the addition of SPO to the feed led to higher yields of olefins for all experiments compared to the benchmarks (and in return, reduced alkane yields). At 550°C, for example, ethylene rose from 3.5 to 4.0 wt%, and propylene increased from 14.6 to 16.5 wt%. This trend was also observed for the butenes and for the experiments at 530°C. The alkanes, on the other hand, decreased from 17.3 to 15.9 wt% and from 15.9 to 14.2 wt% at 550°C and 530°C, respectively.

**Figure 6.**
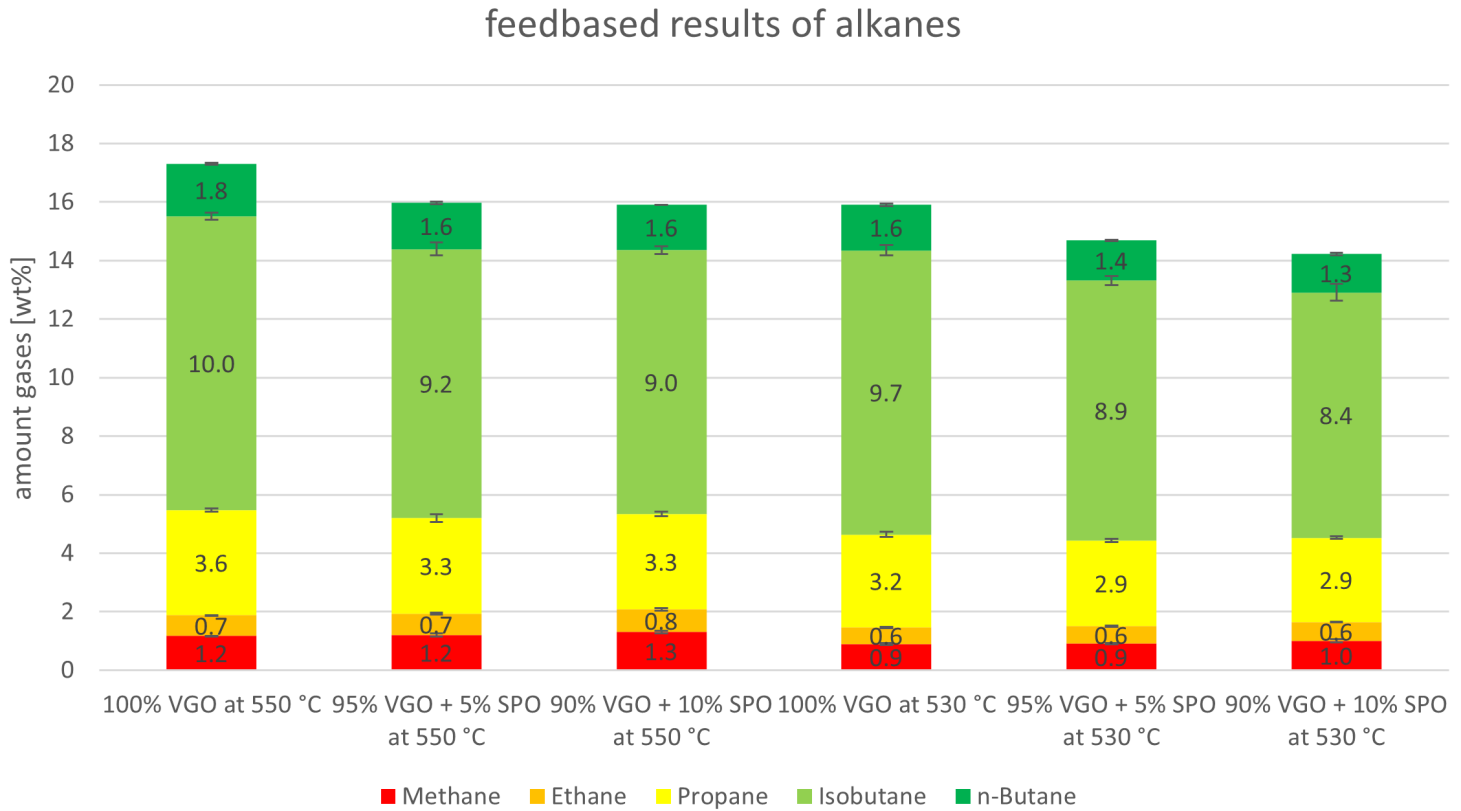
Alkanes in product gas depending on riser temperature and SPO admixture.

The yields of “other gases”, the alkanes, are summarized in more detail in Figure 6. The alkanes consist of methane, ethane, propane, isobutane and n-butane. Independently from the temperature, the amount of methane and ethane was constant for the VGO benchmark and when SPO was co-fed. Values for methane were 1.2 and 0.9 wt% for 550°C and 530°C, respectively. For ethane, they were 0.7-0.8 wt% and 0.6 wt%. A slight decline was observed for propane which dropped from 3.6 to 3.3 wt% at 550°C and from 3.2 to 2.9 wt% at 530°C. Here, the amount of co-feed SPO did not alter the amount of propane. However, a significant decline for isobutane, the biggest alkane product, was observed. Isobutane production declined from 10.0 to 9.0 wt% and from 9.7 to 8.4 wt%, at 550°C and 530°C, respectively. A slight decrease was also observed for n-butane. These changes probably originated from the different compounds in VGO and SPO. For example, VGO contains higher amounts of long-chain paraffins compared to SPO, that can be easily converted into isobutane via cracking and isomerization.

**Figure 7.**
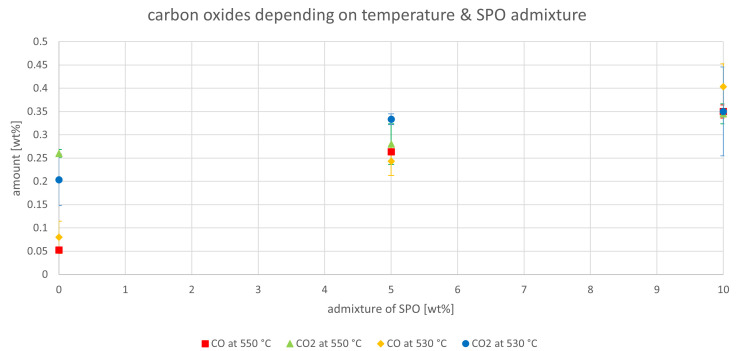
Carbon oxides in product gas depending on Riser-temperature and SPO admixture.

The carbon oxides were analyzed to determine the influence of oxygen in the feed on their formation. The amount of carbon monoxide and dioxide, depending on the amount of SPO in the feed and the riser temperature, are depicted in Figure 7. VGO normally has an oxygen content close to 0, whereas SPO has a significantly higher oxygen content (nearly 40 wt%). Figure 7 shows that carbon oxide content for VGO was not 0 but between 0.05 to 0.25 wt%. This is not necessarily due to oxygen in the VGO but to leakage from the regenerator section, which is fluidized with air. Higher values were obtained for carbon oxides when SPO was co-fed. However, the error margins were often greater than the changes (for example, for CO _2_ at 550°C comparing pure VGO and the admixture of 5 wt% SPO). Nonetheless, a small increase could be observed, justifying the assumption that the oxygen in the SPO partly reacted to carbon oxides. However, these amounts were small compared to the amount of water that was generated (see Figure 3 and Figure 4) which also originates from the oxygen in SPO.

### FCC results for co-feeding of SDPO with VGO

Similar experiments to those with SPO were conducted with SDPO. In the following figures, the same visualization diagrams as with SPO were used to evaluate how the second hydrogenation step of pyrolysis oils reflects onto the product spectra. What can be said up front, is that SDPO showed significantly higher stability during co-feeding, as no clogging of the feeding pipes was observed. This resulted in a more stable plant operation leading to lower error margins.

**Figure 8.**
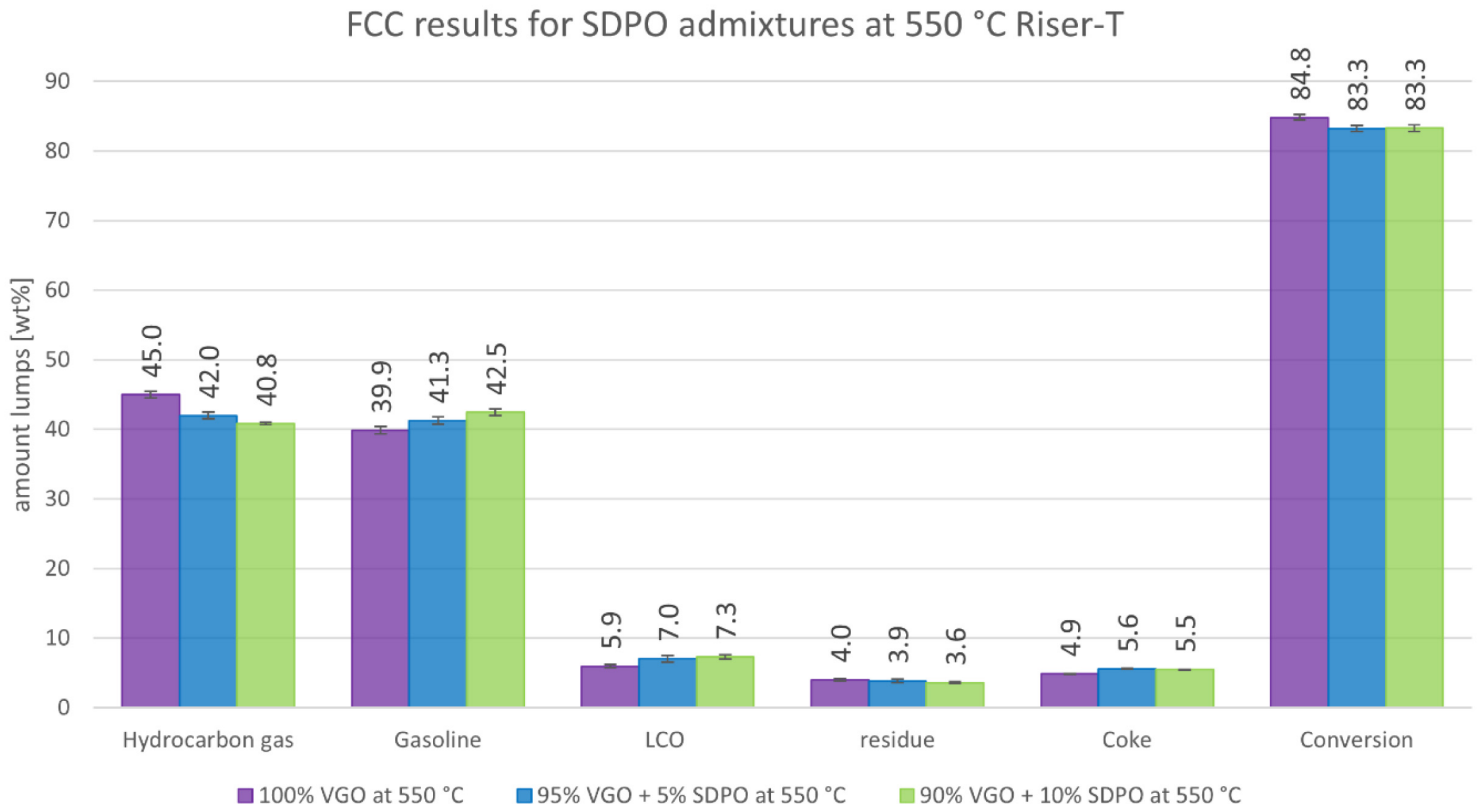
Product distribution depending on SDPO admixture at 550°C Riser-temperature.

Figure 8 summarizes the results of the product lumps depending on the amount of co-fed SDPO at 550°C. As a major contrast to the results with SPO, the co-feeding of SDPO led to an increased production of gasoline and to decreasing gas yields. The gas lump declined from 45.0 to just below 41 wt%, whereas the gasoline amount increased from almost 40 to 42.5 wt%. Additionally, LCO increased slightly from 5.9 to 7.3 wt% and the residue decreased from 4.0 to 3.6 wt%. The coke lump also increased from 4.9 to 5.5 - 5.6 wt%, but was still lower than for SPO. The overall conversion fell slightly from 84.8 to 83.3 wt%. The low amounts of oxygen and water in the SDPO did not lead to a measurable amount of non-dissolved water in the products. Overall, the results were very promising regarding the generation of high-value products, especially of gasoline.

**Figure 9.**
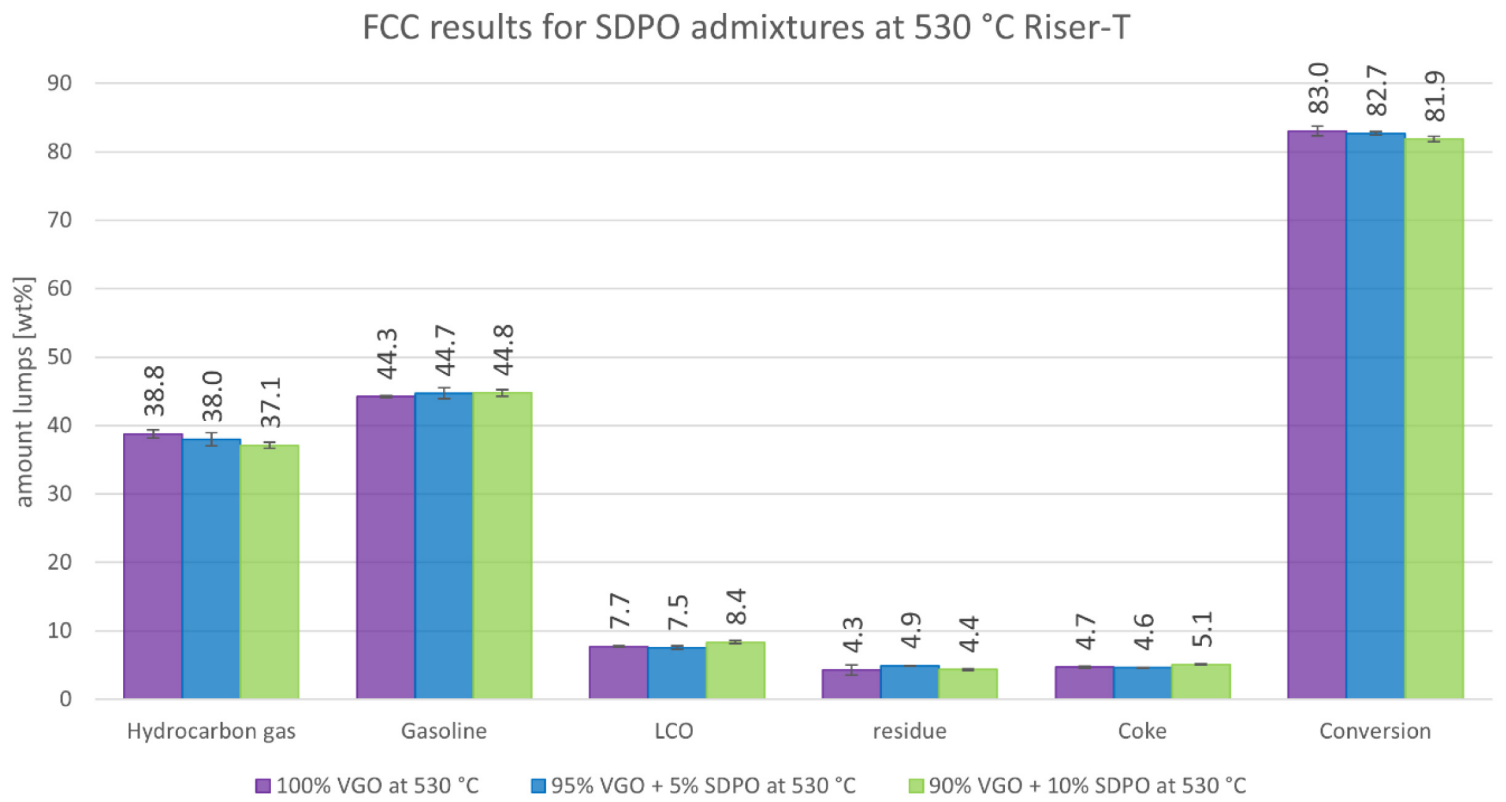
Product distribution depending on SDPO admixture at 530 °C Riser-temperature.

The results obtained at 530°C are illustrated in Figure 9. Again, the amount of gas production was lower while the gasoline generation was higher compared to those at 550°C, which is a general effect of lower riser temperatures. However, the trends showed slight differences. The decline in gas was still significant with a decrease from 38.8 to 37.1 wt%. However, the rise in gasoline production was only slightly higher than the error margins and not as severe as at 550 °C. The LCO production rose from 7.7 to 8.4 wt% while the residue reached its maximum at an admixture of 5 wt% SDPO, with a value of 4.9 wt%. The coke production also increased slightly from 4.7 to 5.1 wt%. The conversion fell only minimally from 83.0 to 81.9 wt%. Again, no measurable amount of non-dissolved water in the products was observed. To summarise, the product spectra were also promising for co-feeding SDPO at 530°C, showing less differences between the benchmark and the co-feeding experiments, compared to the results at 550°C. 

**Figure 10.**
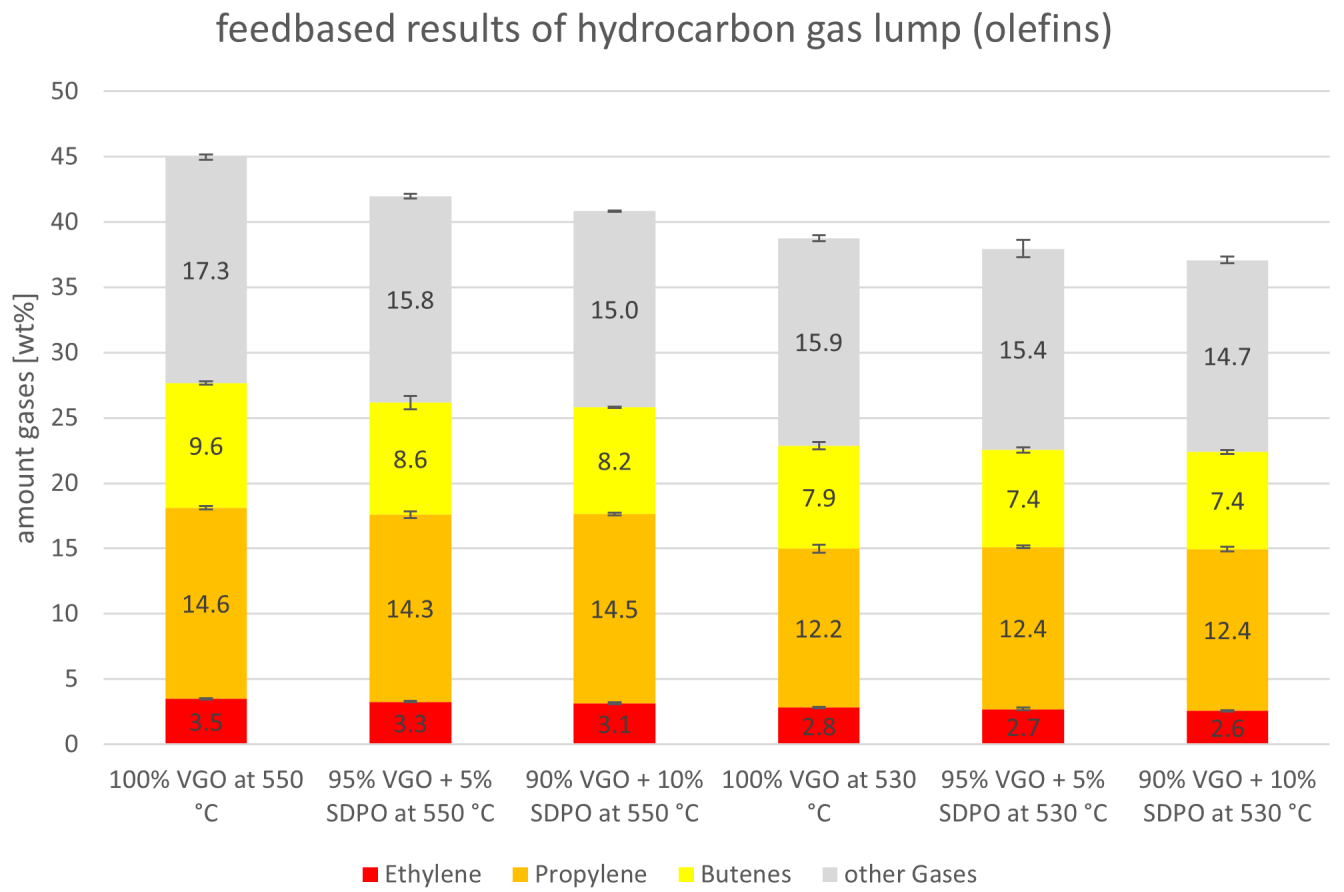
Olefins and other gases (alkanes) depending on Riser-temperature and SDPO admixture.

The gas yield is detailed in Figure 10. For both riser temperatures, the yields are depicted with a focus on olefins (ethylene, propylene and butenes). The “other gases” are alkanes. The ethylene lump shrank slightly when SDPO was co-fed from 3.5 to 3.1 wt% and from 2.8 to 2.6 wt% at 550°C and 530°C, respectively. Propylene, however, was relatively stable between 14.6 and 14.3 wt% at 550°C, and 12.2 and 12.4 wt% at 530°C. The butenes dropped for both riser temperatures with the lowest values being obtained at an admixture of 10 wt% SDPO. Lastly, the other gases also declined from 17.3 to 15.0 wt% and from 15.9 to 14.7 wt% at 550°C and 530°C, respectively. Globally, it is not surprising that most compounds in the gas lump declined since the overall gas lump also declined. Nonetheless, the stable propylene yield, a high-value product used for polymer production, is satisfying since it showed an increase in gas quality. Overall, the riser temperature had a more significant influence than the co-feeding of up to 10 wt% SDPO on the amount of olefins produced.

**Figure 11.**
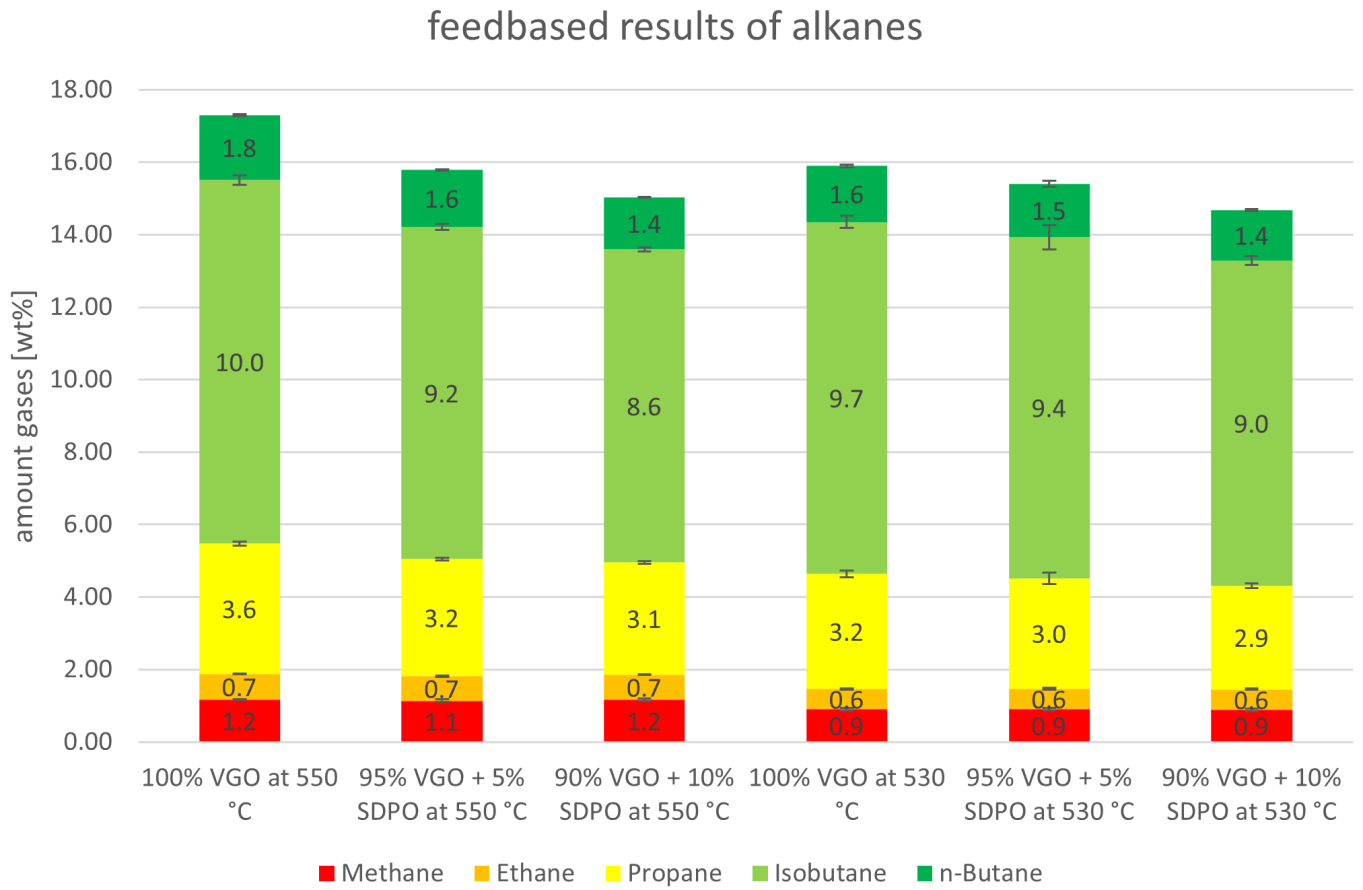
Alkanes in product gas depending on Riser-temperature and SDPO admixture.

Similar to Figure 6, the alkanes for the SDPO co-feed are presented in Figure 11. As with SPO, methane and ethane were stable when SDPO was co-fed. Propane fell slightly from 3.6 to 3.1 wt% at 550°C, and from 3.2 to 2.9 wt% at 530°C. The greatest change was observed for isobutane, for example at 550 °C with a decrease from 10.0 to 8.6 wt%, as it is the largest alkane product lump. This likely originated in the reaction mechanisms that occurred during fluid catalytic cracking, which consist of isomerization reactions in addition to cracking. Lastly, n-butane also showed a falling trend when SDPO was co-fed. Again, the falling trends were expected to some extent, since the total gas lump also showed a falling trend.

**Figure 12.**
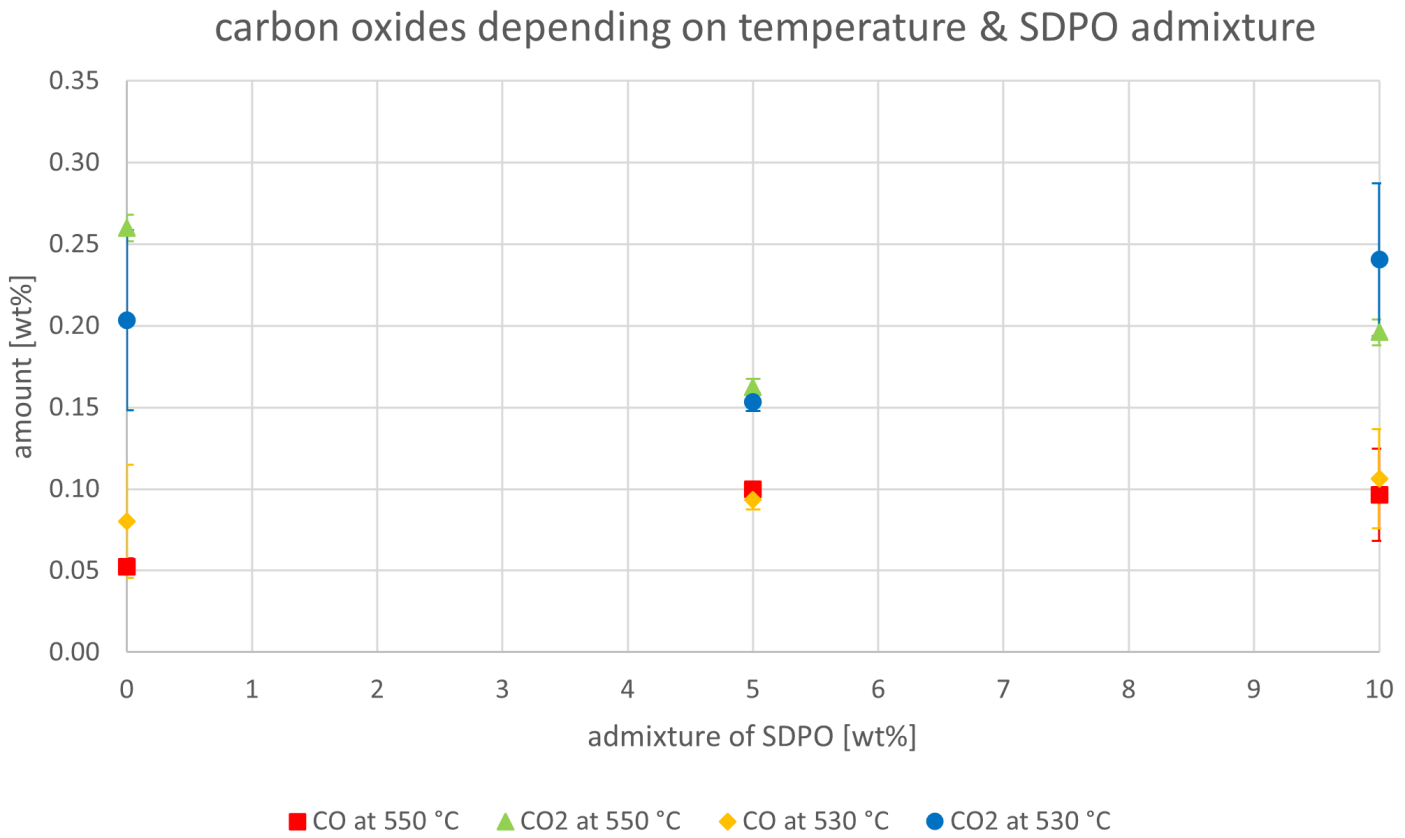
Carbon oxides in product gas depending on Riser-temperature and SDPO admixture.

The effect of the co-feeding of SDPO on the carbon oxides is depicted in Figure 12. There, no clear trends can be observed. The generation of carbon oxide normally originates from the oxygen content in the feedstock; since SDPO has a higher oxygen content than VGO, a possible increase in the formation of carbon oxide could have been expected. The fact that this was not found to be significant leads to the conclusion that the oxygen in the SDPO probably reacted to other products. This is likely due to the much lower oxygen content of SDPO compared to SPO. It is possible that an increase in carbon oxides could be seen when admixtures higher than 10 wt% of SDPO are co-fed.

To summarize, the co-feeding of up to 10 wt% SDPO and SPO led to satisfying results in relation to the gaseous product lumps and their components. No undesirable results are noted for the implementation of SPO or SDPO co-feeding on an industrial level. The coking tendencies of pyrolysis oils in the feeding lines, however, must be addressed, as was already mentioned in previous publications ^8, 17^.

## Gasoline lump

**Figure 13.**
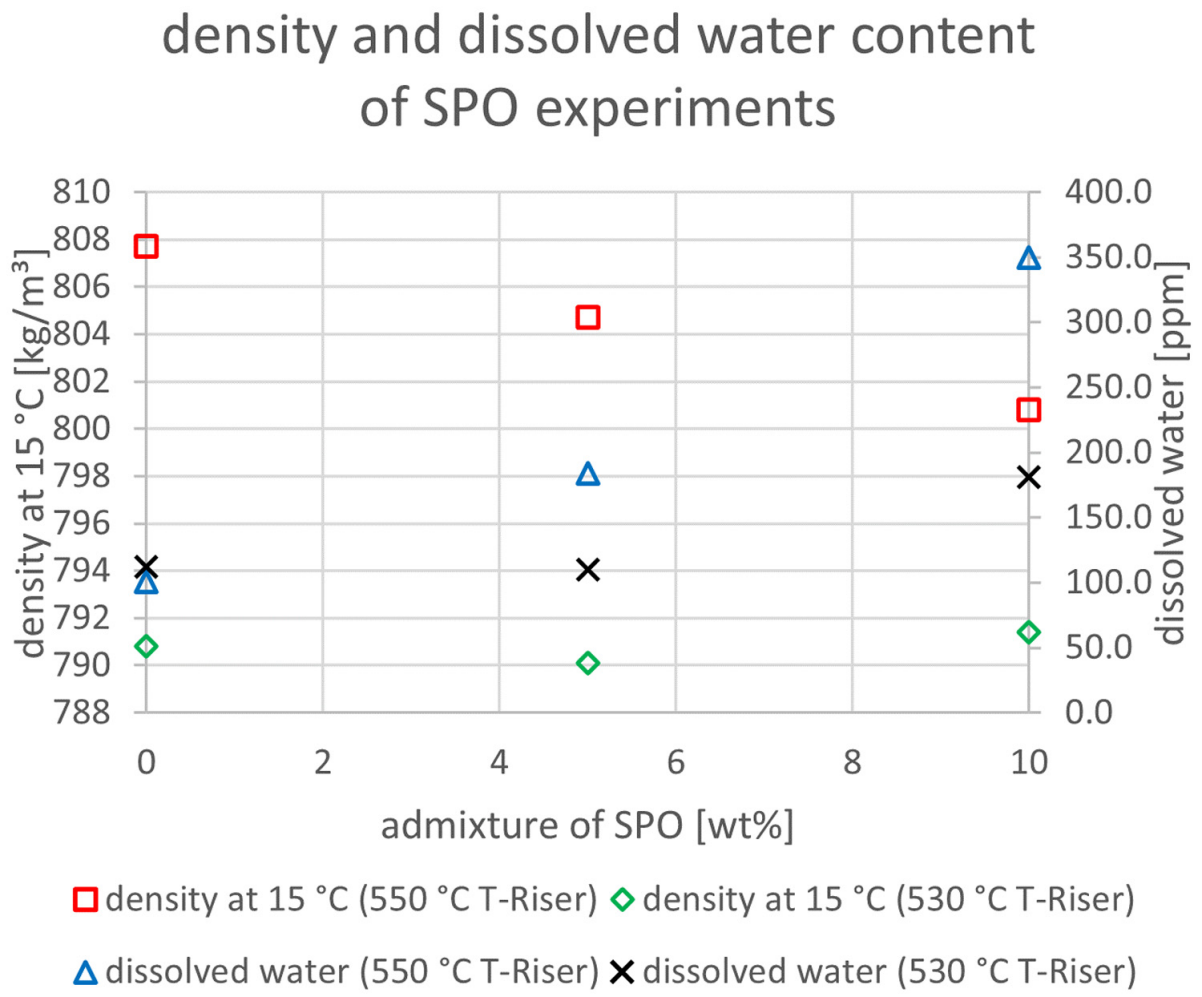
Density and dissolved water content depending on temperature and admixture of SPO.

The relevant parameters chosen to compare the gasoline fractions from the different experiments were density at 15 °C, dissolved water content, nitrogen and sulfur content as well as paraffin, olefin, naphthene and aromatics content. Most other measured components did not show significant changes between the experiments. The results are depicted below ( Figure 13 to Figure 20). In the diagrams below, no error margins are shown since the measurements at the OMV laboratory were only done once.

**Figure 14.**
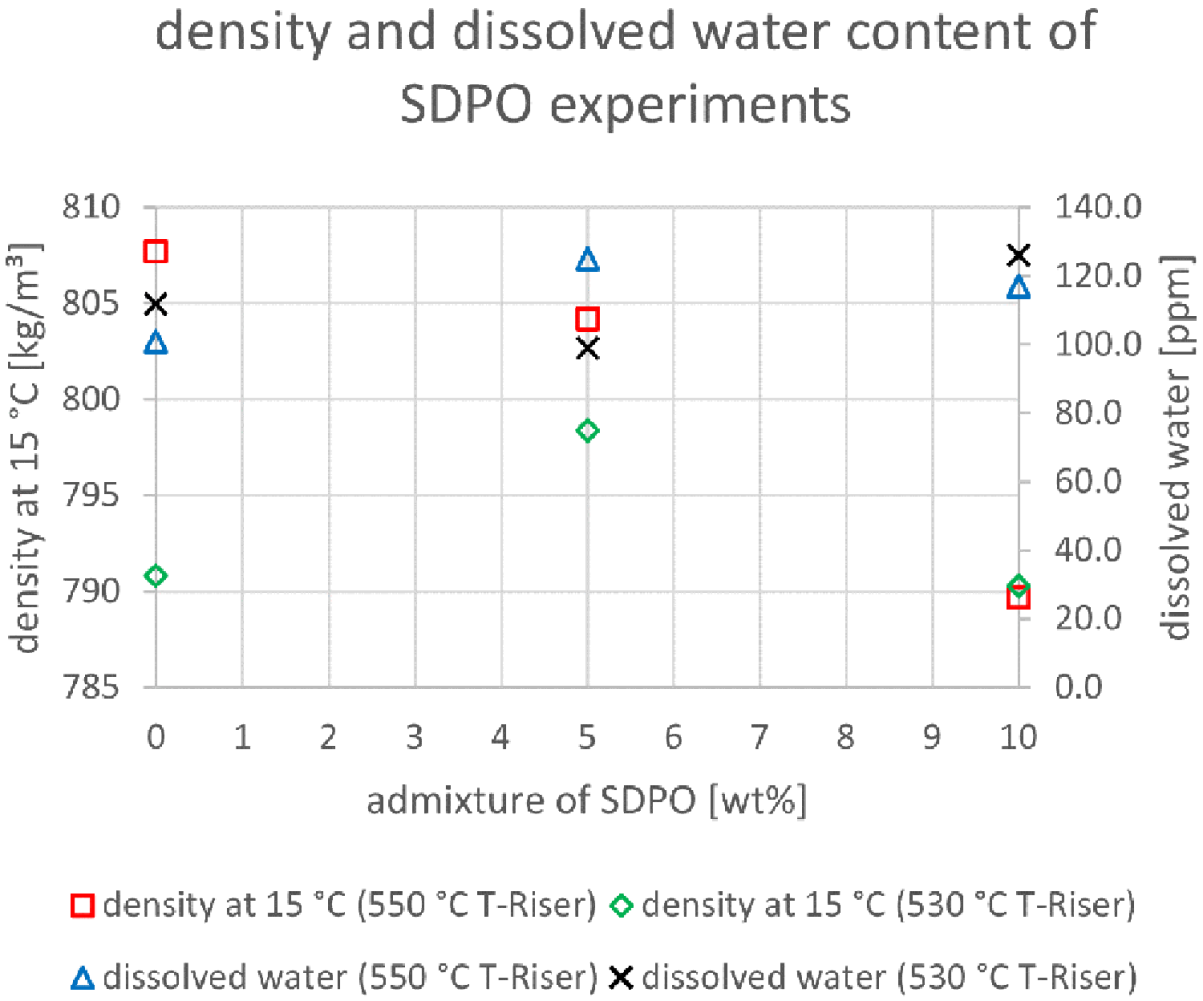
Density and dissolved water content depending on temperature and admixture of SDPO.

**Figure 15.**
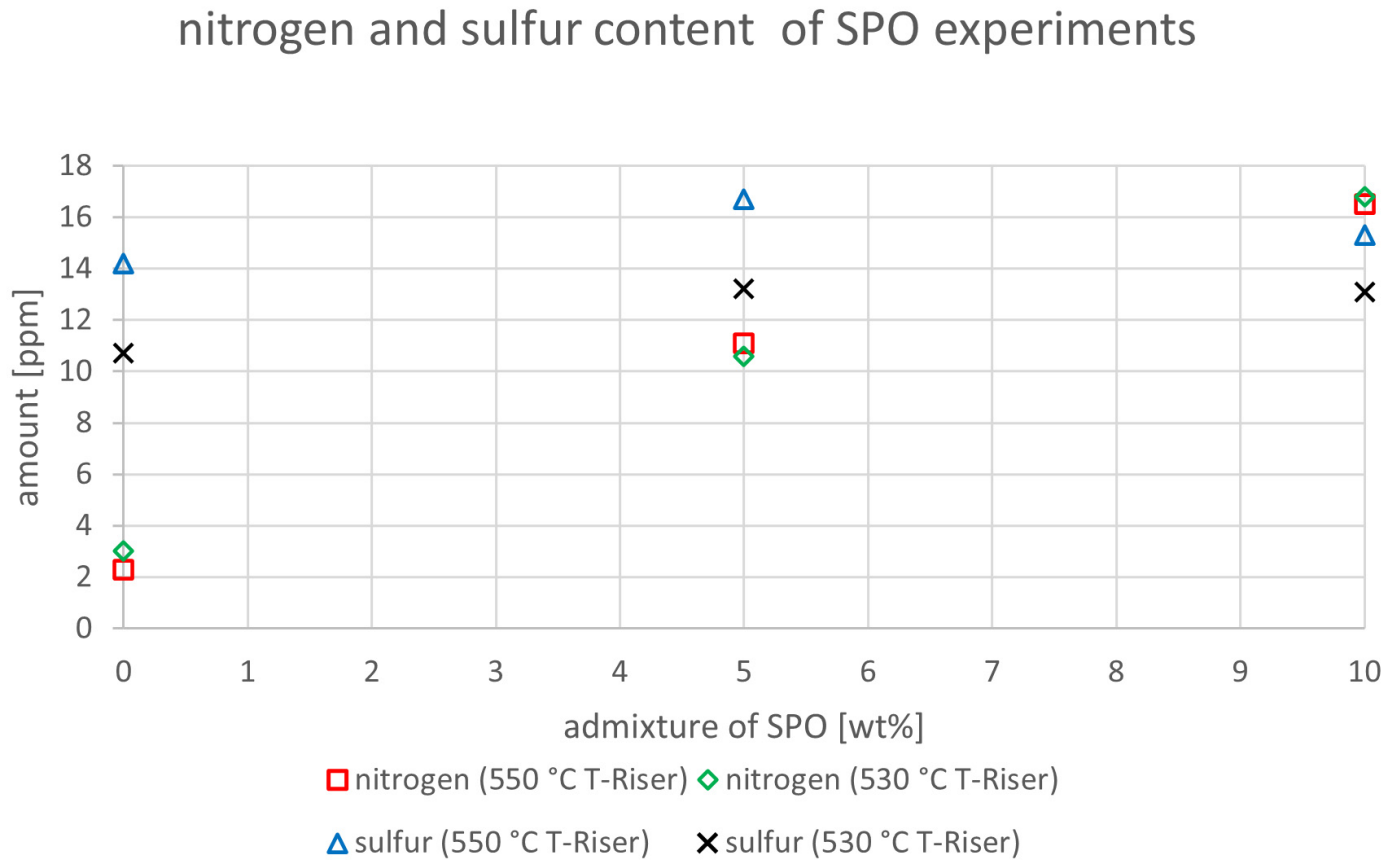
Nitrogen and sulfur content depending on temperature and admixture of SPO.

**Figure 16.**
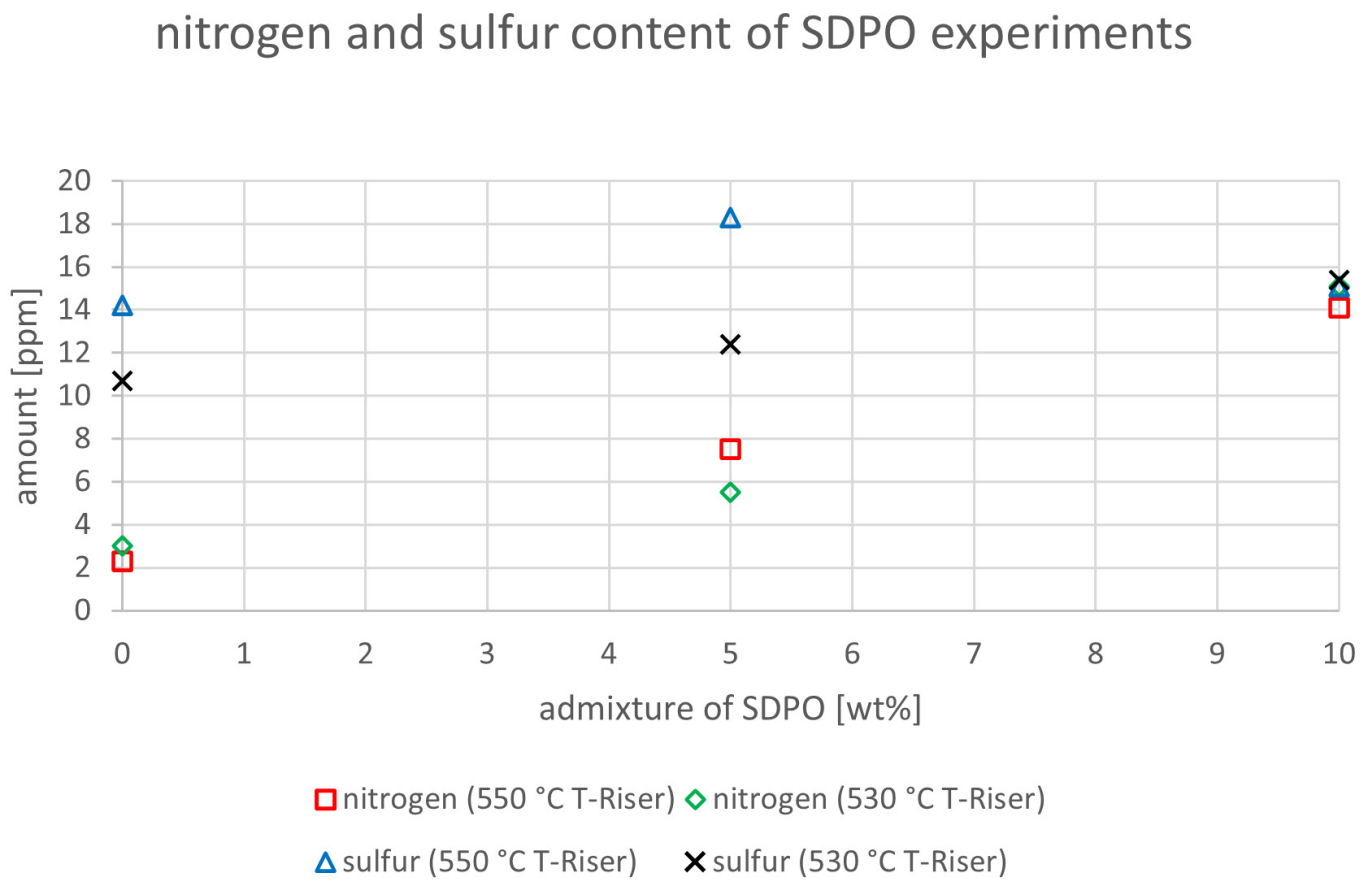
Nitrogen and sulfur content depending on temperature and admixture of SDPO.

**Figure 17.**
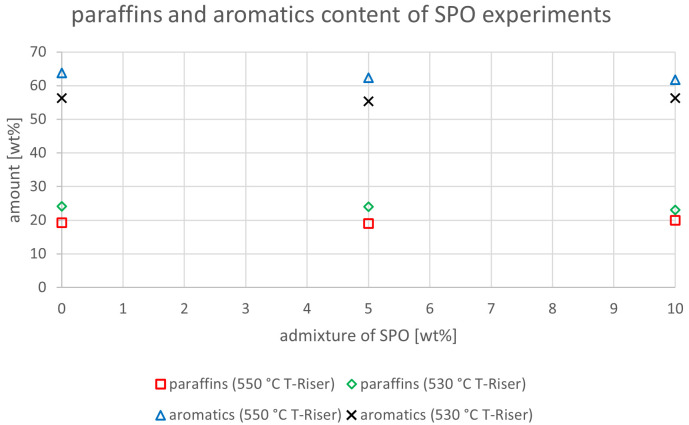
Paraffins and aromatics content depending on temperature and admixture of SPO.

**Figure 18.**
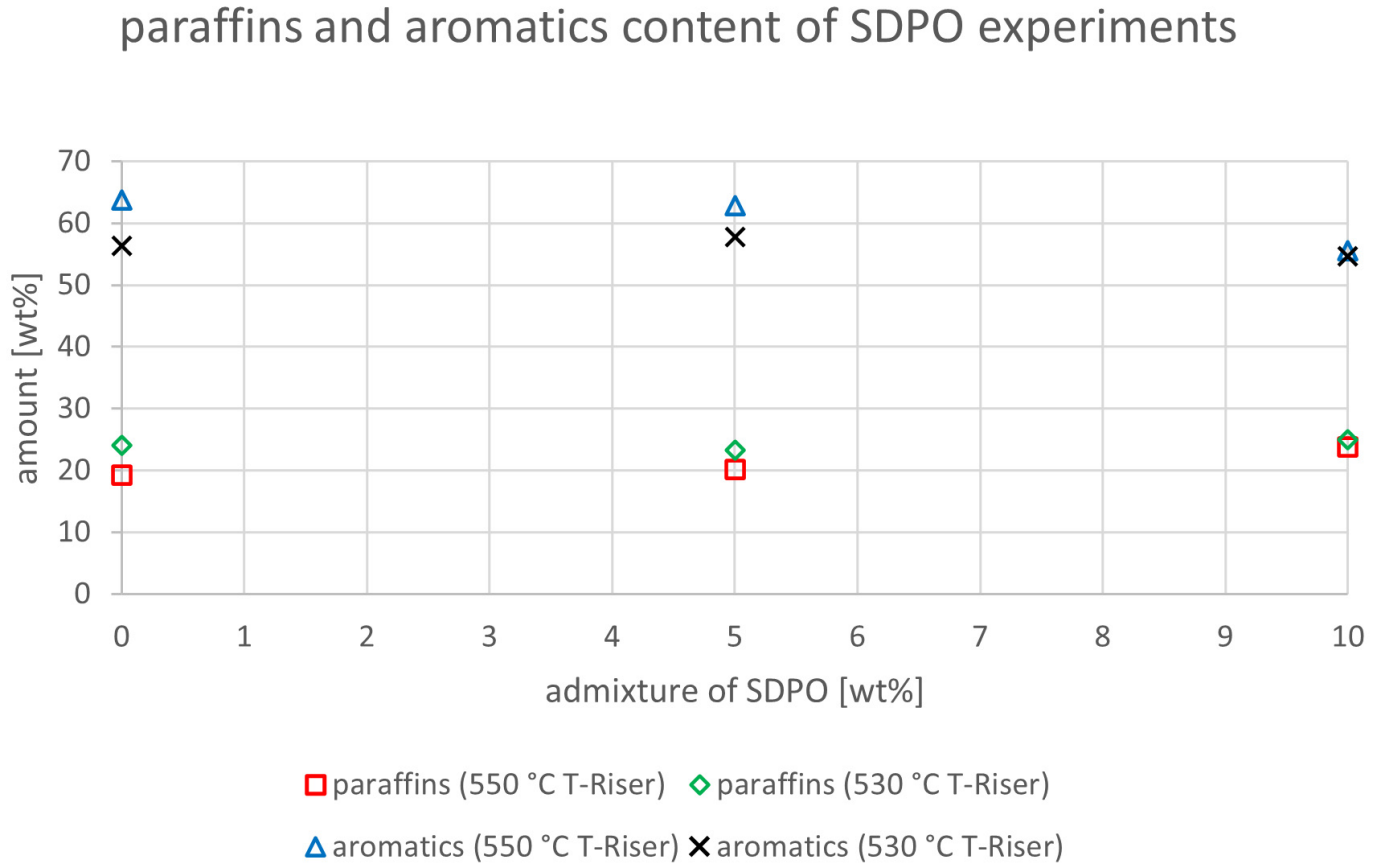
Paraffins and aromatics content depending on temperature and admixture of SDPO.

**Figure 19.**
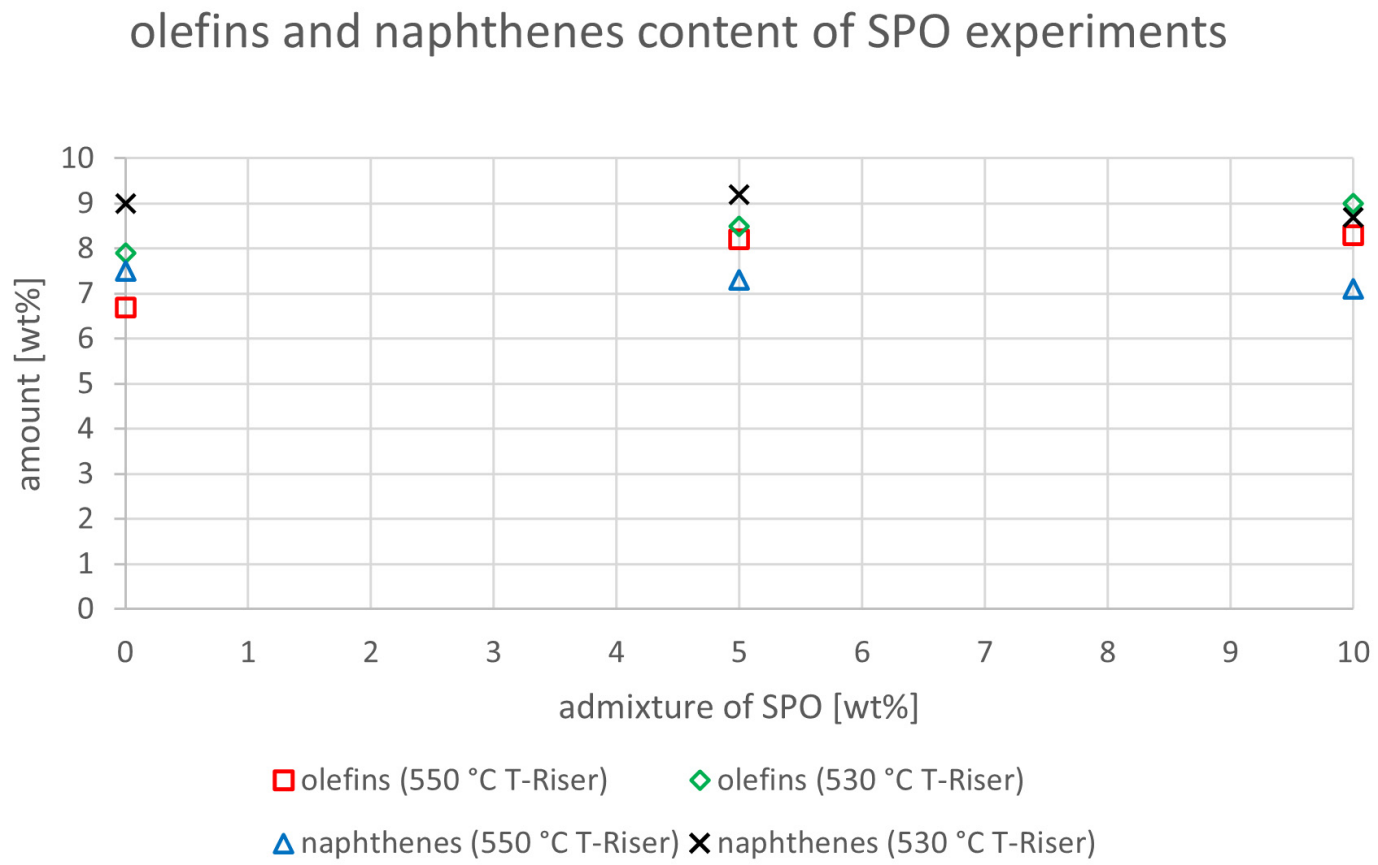
Olefins and naphthenes content depending on temperature and admixture of SPO.

**Figure 20.**
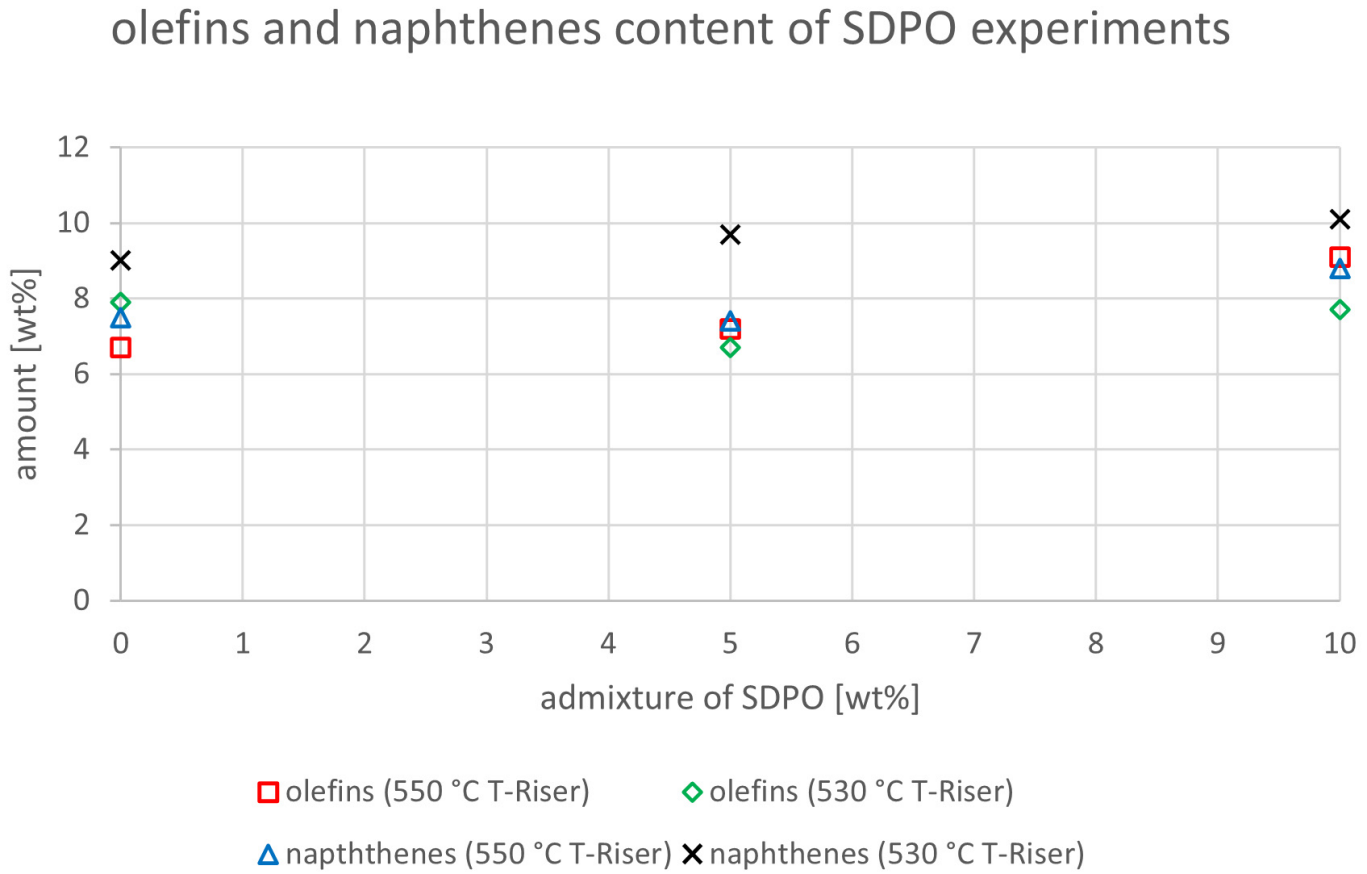
Olefins and naphthenes content depending on temperature and admixture of SDPO.

In Figure 13 the dissolved water content and density at 15°C for SPO are depicted. These parameters were chosen because SPO has a significantly higher amount of water and oxygen than VGO, which could possibly end up in the gasoline. Higher water content would consequently also impact the density. This shows that for both temperatures, the admixture led to higher amounts of dissolved water content. However, this effect was observed more strongly at 550°C with a rise from 101 to 350 ppm. The change at 530°C was a significantly less sharp increase from 112 to 181 ppm. Interestingly, the higher water content at 550°C occurred together with a decrease in density from 807.7 to 800.8 kg/m³ (since water has a higher density than gasoline, a rise could be expected). The reason for this is probably that other changes in composition had a stronger impact on density than the still comparably low dissolved water content. The density at a 530°C riser temperature was rather constant with values around 790 kg/m³. Additionally, a lower riser temperature led to lower densities and to mostly lower contents of dissolved water.

In Figure 14 the same parameters as in Figure 13 are illustrated, but for SDPO. The dissolved water content showed a much more constant value than for SPO. At 550°C it stayed relatively stable between 100 and 125 ppm. The values at 530 °C showed the same trend. Thus, the lower water content and oxygen content in SDPO compared to SPO led to significantly less water in the gasoline. The density at 550 °C riser temperature showed a falling trend at higher SDPO ratios (807.7 to 789.7 kg/m³) whereas the values at 530°C fluctuated between 790 and 798 kg/m³. The values for density were comprised between 790 and 810 kg/m³ for all experiments, independent of whether SPO or SDPO was co-fed. All these densities, however, were too high, since gasoline must have a density between 720 and 775 kg/m³ as described in DIN EN 228:2017 ^15^, which must be taken into account during blending.

The differences between carbon and hydrogen content were insignificant and are not graphically illustrated in this paper. Besides, the oxygen content in all gasoline samples was below 0.1 wt% and fulfils the standards ^15^. However, realizing that biogenic feedstocks often have significantly higher nitrogen and sulfur contents than hydrogenated fossil feedstocks, these two contaminants were analyzed in further detail ( Figure 15). The sulfur content showed an indifferent trend with values varying between 14.2 and 16.7 ppm at 550°C, and between 10.7 and 13.2 ppm at 530°C. All these values exceed the requirements defined by European norms at 10 ppm ^15^. Nonetheless, these results are promising since they do not show a significant impairment of quality when SPO was co-fed. This was partly expected since SPO is hydrogenated like VGO, and hydrogenation is an often-used method to desulfurize feedstocks.

The results for nitrogen, however, showed an increase with the highest value being obtained at admixtures of 10 wt% SPO. This is due to the higher content of nitrogen in SPO compared to VGO. The increment was from 2.3 to 16.5 ppm at 550°C, and from 3 to 16.8 ppm at 530°C.

The same diagram was also created for SDPO (see Figure 16). Analogue to Figure 15, the experiments with SDPO also showed indifferent trends regarding sulfur content. While at 530°C the sulfur amount increased from 10.7 to 15.4 ppm, at 550°C it varied between 14.2 to 18.3 ppm. Experiments at lower riser temperatures led to lower sulfur contents in the gasoline, an outcome that was also observed when SPO was co-fed. However, all values were once again above the limit value defined by European norms ^15^. The nitrogen content showed the same trend as depicted in Figure 15 for SPO. Higher admixtures of SDPO led to higher nitrogen contents in the gasoline. The highest values were obtained at 10 wt% SDPO admixture, with 14.1 ppm at 550°C and 15.1 ppm at 530°C. These values were slightly lower than when SPO was co-fed, but nonetheless show an increase compared to the VGO base case. Additionally, the heating value was calculated according to the Boie formula ^18^. However, no significant differences were observed with values between 41.4 and 41.8 MJ/kg. This was expected due to the low changes in the elemental composition of the different gasoline samples.

Finally, in the following four diagrams, the content of paraffins, aromatics, olefins and naphthenes are depicted. In Figure 17, the paraffin contents at 550°C and 530°C were constant with values around 19-20 and 23-24 wt%, respectively. The aromatics content slightly decreased from 63.8 to 61.7 wt% at 550 °C, but varied between 56.3 and 55.3 wt% at 530 °C. Both values for aromatics and paraffins were higher under lower riser temperatures. A limit value does not exist for paraffins, however one exists for aromatics at 35 vol% ^15^. All obtained gasoline samples were significantly above this value, which must be considered at blending. The reason for these high aromatics in the gasoline fraction could be the fact that FCC units are not able to significantly crack aromatic rings, which then pass through the reactor section and enrich the liquid product phase in these compounds.

The paraffin and aromatics content for the SDPO admixtures were slightly higher than the ones for SPO ( Figure 18). The values for 550°C showed a rising trend from 19.3 to 23.8 wt%, while no clear trend was shown for 530°C with values between 23.3 and 25 wt%. The aromatic compounds showed a similar trend as the paraffins, with a clear decline at 550°C from 63.8 to 55.6 wt% and varying numbers between 57.8 and 54.6 wt% at 530 °C. Again, the values obtained for paraffins were lower at higher temperatures and the aromatics content was greater at higher riser temperatures. Again, the aromatics content was above the limiting value.

The olefin and naphthene contents of the SPO tests are illustrated in Figure 19. For 550°C and 530 °C, the olefin content rose from 6.7 to 8.3 wt% and from 7.9 to 9.0 wt%, respectively. All these values were below the limit value of 18 v% as defined in 15. The naphthene content slightly decreased from 7.5 to 7.1 wt% at 550 °C, but showed no clear trend with values between 8.7 and 9.2 wt% at 530 °C. The same components for the SDPO testing campaign are shown in Figure 20. Here, all olefin contents were below the defined limiting value ^15^ as well. At 550°C, olefins rose from 6.7 to 9.1 wt% and varied between 6.7 and 7.9 wt% at 530°C. The naphthenes increased at both temperatures from 7.5 to 8.8 wt% and from 9 to 10.1 wt%, at 550°C and 530°C, respectively. For both experimental designs, the temperature had a higher influence on the creation of naphthenes in the gasoline than the admixture of up to 10 wt% SPO or SDPO.

In this work, the co-feeding of up to 10 wt% SPO and SDPO, together with VGO, was accomplished in a continuously operated FCC pilot plant. SDPO was mixed beforehand with VGO and fed via one pump into the riser section of the plant. The SPO, however, was not completely mixable with VGO and therefore a two-pump feeding system was established. The two feeds got in contact before the tubular oven and were fed passing said oven into the riser via one pipe. This feeding system worked, but at a 10 wt% admixture of SPO, plant operation instabilities were observed, which led to higher error margins in the results. These instabilities were caused by clogging in the feeding pipes that was generated by the partial coking of the SPO due to the heat. As a consequence, a different feeding system should be chosen for higher admixtures of SPO, preferably a completely separate feeding of SPO and VGO. SDPO did not show any signs of clogging, and therefore, a one-pipe feeding solution suffices judging from these experiments.

The co-feeding of up to 10 wt% SPO led to a change in the gas production from 45.0 to 46.3 wt%, and from the gasoline production from 39.9 to 36.8 wt% at 550°C. The change at 530°C was less drastic, but the overall tendencies were still similar. However, as mentioned before, the experiments with 10 wt% SPO, especially at 530°C, had high error margins; therefore experiments at higher admixtures and with a different feeding system are advised to further research the trends when SPO is co-fed. The amount of olefins produced was positively influenced by the co-feeding of SPO, with a rise in propylene at 550°C from 14.6 to 16.5 wt%, for example. Overall conversion rates dropped only slightly from 84.8 to 83.1 wt%, and from 83.0 to 81.1 wt% at 550°C and 530°C, respectively. So, it can be concluded that no significant negative product shift occurs when co-feeding up to 10 wt% SPO. Judging from the results, SPO is more suitable as a feedstock when the focus of the FCC plant is on gas, and especially on olefin production for which the results were even more promising. Judging by these positive results, with a different feeding system, higher substitution rates should be possible. As a first step substitution in the range of 20 – 30 wt% is recommended, which should be tested in further experiments.

The experiments with SDPO showed different outcomes than their counterparts with SPO, as they led to different product shifts. The admixture of up to 10 wt% SDPO led to a higher generation of gasoline (39.9 to 42.5 wt%) and a lower generation of gas (45.0 to 40.8 wt%) at 550°C. The same trends were observed at a 530°C riser temperature but less drastically. The overall conversion only showed a small decline from 83.0 to 81.9 wt%. The valuable gas components, namely olefins, also slightly declined, with the exception of propylene that remained stable around 14.3 to 14.6 wt%, and 12.2 to 12.4 wt%, at 550°C and 530°C respectively. Unlike with SPO, no water formation was observed (compared to up to 1.8 wt% at an admixture of 10 wt% SPO at 530°C). So, regarding the goal of the Waste2Road project, which is the generation of waste-derived biogenic fuels, the highest yield of gasoline was obtained with admixtures of SDPO at 530°C riser temperature. Judging from these results, even a 100 wt% SDPO feeding seems achievable, which should be tested in further experiments.

The generated liquid products were further separated by manual distillation, and gasoline samples were obtained. These samples were sent to OMV for analysis. The sulfur and nitrogen contents of the gasoline samples were measured to determine possible increases that possible originate from sulfur and nitrogen traces in the pyrolysis oils. Sulfur hereby did not show a significant rise, which is desireable since low limit values (10 ppm) are dictated by European norms ^15^. However, all samples (including the ones generated using only pure VGO) were slightly above said limit value. The highest value was obtained at 18.3 ppm when SDPO was co-fed at 530°C. No clear difference was observed between the SPO and SDPO samples, which is interesting since SDPO went through a second hydrogenation step, and hydrogenation is often used to desulfurize feedstocks. The nitrogen content, however, revealed the clear tendency that using pyrolysis oils lead to increased amounts of nitrogen in the gasoline compared to pure VGO. The results from the SPO and SDPO experiments both showed that higher amounts of pyrolysis oils lead to higher amounts of nitrogen in the feedstock. This probably originates from the significantly higher nitrogen content in the pyrolysis oils compared to the fossil VGO feedstock. While these values were still quite low with a maximum of around 16-17 ppm, the possible higher formation of nitrogen oxides (higher fuel NO) through combustion should still be taken into consideration ^19^. Nonetheless, no alarming signs were found that would prohibit the obtained gasoline samples to be used as blending components to generate gasoline that would fulfill all requirements defined by European norms ^15^.

## Data availability

### Underlying data

Zenodo: Data - Co-feeding of VGO and pine-wood derived hydrogenated pyrolysis oils in an Fluid Catalytic Cracking pilot plant to generate olefins and gasoline https://doi.org/10.5281/zenodo.5568908 ^20^

## References

[1] UNFCCC: Report of the Conference of the Parties on its twenty-first session, held in Paris from 30 November to 13 December 2015 Addendum Contents Part two: Action taken by the Conference of the Parties at its twenty-first session. *Decis 1/CP.21 Adopt. Paris Agreem.* 2015;01192:1–36. Reference Source

[2] European Commission: A Clean Planet for all. A European long-term strategic vision for a prosperous, modern, competitive and climate neutral economy. 2018. Reference Source

[3] Akporiaye D : WASTE2ROAD - Biofuels from WASTE TO ROAD transport. 2020; [Accessed: 14-Dec-2020]. Reference Source

[4] Sadeghbeigi R : Chapter 1 - Fluid catalytic cracking process description—converter section.In *Fluid Catalytic Cracking Handbook.* Fourth., Butterworth-Heinemann,2020;1–22. 10.1016/B978-0-12-812663-9.00001-1

[5] Büchele M Swoboda M Reichhold A : Canola oil/glycerol mixtures in a continously operated FCC pilot plant and comparison with vacuum gas oil/glycerol mixtures. *Chem Eng Process. - Process Intensif.* 2019;142:107553. 10.1016/j.cep.2019.107553

[6] Melero JA García A Clavero M : Production of biofuels via catalytic cracking.Woodhead Publishing Limited, 2011;5(3):390–419. 10.1533/9780857090492.3.390

[7] Malleswara Rao TV Dupain X Makkee M : Fluid catalytic cracking: Processing opportunities for Fischer-Tropsch waxes and vegetable oils to produce transportation fuels and light olefins. *Microporous Mesoporous Mater.* 2012;164:148–163. 10.1016/j.micromeso.2012.07.016

[8] de Rezende Pinho A de Almeida MBB Mendes FL : Fast pyrolysis oil from pinewood chips co-processing with vacuum gas oil in an FCC unit for second generation fuel production. *Fuel.* 2017;188:462–473. 10.1016/j.fuel.2016.10.032

[9] de Rezende Pinho A de Almeida MBB Mendes FL : Co-processing raw bio-oil and gasoil in an FCC Unit. *Fuel Process Technol.* 2015;131:159–166. 10.1016/j.fuproc.2014.11.008

[10] Gueudré L Thegarid N Burel L : Coke chemistry under vacuum gasoil/bio-oil FCC co-processing conditions. *Catal Today.* 2015;257 part 2:200–212. 10.1016/j.cattod.2014.09.001

[11] Gueudré L Chapon F Mirodatos C : Optimizing the bio-gasoline quantity and quality in fluid catalytic cracking co-refining. *Fuel.* 2017;192:60–70. 10.1016/j.fuel.2016.12.021

[12] Reichhold A : Entwicklung von Reaktions/Regenerationssystemen für Adsorptions/Desorptionsprozesse und für katalytisches Cracken auf Basis von intern zirkulierenden Wirbelschichten.TU Wien, 1996.

[13] Bielansky P : Alternative Feedstocks in Fluid Catalytic Cracking.TU Wien, 2012. Reference Source

[14] Fahim MA Alsahhaf TA Elkilani A : Fundamentals of Petroleum Refining. 2010. 10.1016/C2009-0-16348-1

[15] DIN Deutsches Institut für Normung: Kraftstoffe – Unverbleite Ottokraftstoffe – Anforderungen und Prüfverfahren; Deutsche Fassung EN 228: 2012+A1: 2017.Berlin, 2017. Reference Source

[16] Laredo GC Vega Merino PM Hernández PS : Light Cycle Oil Upgrading to High Quality Fuels and Petrochemicals: A Review. *Ind Eng Chem Res.* 2018;57(22):7315–7321. 10.1021/acs.iecr.8b00248

[17] Wang C Venderbosch R Fang Y : Co-processing of crude and hydrotreated pyrolysis liquids and VGO in a pilot scale FCC riser setup. *Fuel Process Technol.* 2018;181:157–165. 10.1016/j.fuproc.2018.09.023

[18] Boie W : Beiträge zum feuerungstechnischen Rechnen. *Wissenschaftliche Zeitschrift der Tech. Hochschule Dresden.* 1952;2:53.

[19] Toof JL : A Model for the Prediction of Thermal, Prompt, and Fuel NO _x_ Emissions From Combustion Turbines. *J Eng Gas Turbines Power.* 1986;108(2):340–347. 10.1115/1.3239909

[20] Büchele M : Data - Co-feeding of VGO and pine-wood derived hydrogenated pyrolysis oils in an Fluid Catalytic Cracking pilot plant to generate olefins and gasoline. 2021. 10.5281/ZENODO.5568908 PMC10446091 37645116

