# Supplementary material for: Co-feeding of vacuum gas oil and pinewood-derived hydrogenated pyrolysis oils in a fluid catalytic cracking pilot plant to generate olefins and gasoline
Source: Open Res Eur. 2021 Nov 30;1:143. [Version 1] doi: 10.12688/openreseurope.14198.1 (PMC10446091; doi:10.12688/openreseurope.14198.1)
Supplement: Peer review history

# Peer review: Reviewer response for version 1

**DOI:** 10.21956/openreseurope.15311.r28108

**Published:** 2021-12-20

**Authors:** Kimberly Magrini-Bair

**Affiliations:** 1 Catalytic Carbon Transformation and Scale-up Center, National Renewable Energy Laboratory, Golden, CO, USA

This is a well written and comprehensive article that is relevant to the co-processing community comprising researchers through refinery users. The work details the co-processing of waste-wood derived pyrolysis oils of varied oxygenate content with vacuum gas oil (VGO) using a novel riser reactor system capable of FCC operation with zeolite catalysts comprising equilibrium catalyst. Process data and product compositions and yields are provided and generally agree with literature data on co-processing biomass derived liquids with VGO in riser reactors. This work is of significant interest to researchers.

 Questions:

 Is this novel riser system scalable with refinery FCC units?

 What is the composition of the SPO and SPDO oils with respect to both hydrocarbons and oxygenates?

 What is the biomass feedstock source and composition including contaminants that could be carried into the bio oils?

 Can information on cost to produce the bio oils be included?

 Maybe Criteria

 Information on catalyst, analysis techniques can not be included due to proprietary considerations.

 Some experiments could only be conducted once which does not allow statistical analysis.

Is the study design appropriate and does the work have academic merit?

Yes

Is the work clearly and accurately presented and does it cite the current literature?

Yes

If applicable, is the statistical analysis and its interpretation appropriate?

Yes

Are all the source data underlying the results available to ensure full reproducibility?

Partly

Are the conclusions drawn adequately supported by the results?

Yes

Are sufficient details of methods and analysis provided to allow replication by others?

Yes

Reviewer Expertise:

We currently work in assessing catalyst and bio oil composition impact on FCC co-processing petroleum feedstocks using an industrial compatible Davison Circulating Riser (DCR) reactor.  We additionally are developing catalytic fast pyrolysis oils of low oxygenate content for co-processing.

I confirm that I have read this submission and believe that I have an appropriate level of expertise to confirm that it is of an acceptable scientific standard.

# Peer review: Reviewer response for version 1

**DOI:** 10.21956/openreseurope.15311.r28109

**Published:** 2021-12-15

**Authors:** Daniele Castello

**Affiliations:** 1 Department of Energy Technology, Aalborg University, Aalborg, Denmark

The present manuscript concerns the co-feeding of VGO with two different types of hydrogenated pyrolysis oil from pinewood in a fluid catalytic cracking plant. Tests involved stabilized pyrolysis oil, i.e. oil that underwent mild hydrogenation to remove the most reactive functionalities, and stabilized-deoxygenated pyrolysis oil, where hydrotreatment was conducted in a more severe fashion to remove larger amounts of oxygen. Results showed that both co-feeding options are viable with a blending of 10%, although stabilized pyrolysis oil led to some clogging issues. However, in both cases, a rise in the level of nitrogen in the produced gasoline was observed.

 The work is of good quality and the results are very interesting to the field. The authors reported a large number of analyses and clarified the effect of co-feeding pyrolysis oil.

 Only a few minor remarks can be placed:

1. It is not very clear how the cracking and combustion zones of the FCC were kept separated. Some results actually suggest that oxygen is present in the cracking zone, where it forms carbon oxides.
2. The stabilization of bio-oil was done using four catalytic reactors. However, details are missing on which are the characteristics of these 4 beds, for instance: was it the same catalyst? At which temperature was each reactor kept? Were they operated in series or in parallel?
3. Why was the temperature increase observed only in the fourth reactor?
4. Indication of the space velocity during hydrotreatment/stabilization could be helpful.
5. At page 8, change “conversation” in “conversion”.
6. What is the residence time of the catalyst in the regeneration section of the FCC plant?
7. It should be stated what is the standard followed for SimDist.
8. In order to better evaluate the process, it would be advisable to include also the SimDist results for the stabilized and deoxygenated bio-oils. This will help evaluating the contribution of the renewable feed to the different produced fractional cuts.
9. Graphs are sometimes not very clear. Perhaps adding some trendlines or joining the points with dashed lines could be helpful.

Is the study design appropriate and does the work have academic merit?

Yes

Is the work clearly and accurately presented and does it cite the current literature?

Yes

If applicable, is the statistical analysis and its interpretation appropriate?

Yes

Are all the source data underlying the results available to ensure full reproducibility?

Yes

Are the conclusions drawn adequately supported by the results?

Yes

Are sufficient details of methods and analysis provided to allow replication by others?

Yes

Reviewer Expertise:

biomass; hydrothermal processes; hydrothermal liquefaction; hydroprocessing

I confirm that I have read this submission and believe that I have an appropriate level of expertise to confirm that it is of an acceptable scientific standard.
